# A European multinational cost-effectiveness analysis of empagliflozin in heart failure with reduced ejection fraction

**DOI:** 10.1007/s10198-022-01555-6

**Published:** 2022-12-04

**Authors:** Ali Tafazzoli, Odette S. Reifsnider, Leana Bellanca, Jack Ishak, Marc Carrasco, Pal Rakonczai, Matthew Stargardter, Stephan Linden

**Affiliations:** 1 Evidera, 7101 Wisconsin Avenue, Suite 1400, Bethesda, MD 20814 USA; 2 Boehringer Ingelheim Ltd., Ellesfield Avenue, Bracknell, Berkshire, RG12 8YS UK; 3 Boehringer Ingelheim España S.A, Prat de la Riba 50, 08204 Sant Cugat del Vallès, Spain; 4 Evidera, Dorottya Udvar, Bocskai út 134-146-E épület 2. Emelet, Magyarország, 1113 Budapest, Hungary; 5 Boehringer Ingelheim International GmbH, Binger Str. 173, 55216 Ingelheim am Rhein, Germany

**Keywords:** Cost-effectiveness, Empagliflozin, Heart failure, Hospitalisation, Reduced ejection fraction, Sodium–glucose cotransporter-2 inhibitor, C69, I19

## Abstract

**Purpose:**

This research examined the cost-effectiveness of adding empagliflozin to standard of care (SoC) compared with SoC alone for treatment of heart failure with reduced ejection fraction (HFrEF) from the perspective of healthcare payers in the United Kingdom (UK), Spain and France.

**Methods:**

A lifetime Markov cohort model was developed to simulate patients’ progression through health states based on Kansas City Cardiomyopathy Questionnaire Clinical Summary Score. The model predicted risk of death, hospitalisation for worsening heart failure (HHF), treatment-related adverse events, and treatment discontinuation each monthly cycle. Clinical inputs and utilities were derived from EMPEROR-Reduced trial data, supplemented by published literature and national costing databases. Costs (2021 pound sterling/euro) and quality-adjusted life-years (QALYs) were discounted annually for the UK (3.5%), Spain (3.0%) and France (2.5%).

**Results:**

In the UK, Spain and France, empagliflozin plus SoC yielded additional QALYs (0.19, 0.23 and 0.21) at higher cost (£1185, €1770 and €1183 per patient) than SoC alone, yielding incremental cost-effectiveness ratios of £6152/QALY, €7736/QALY and €5511/QALY, respectively. Reduced HHF incidence provided most cost offsets for empagliflozin plus SoC. Similar results were obtained for a range of subgroups and sensitivity analyses. Probabilistic sensitivity results indicated empagliflozin plus SoC remained cost-effective vs. SoC at willingness-to-pay thresholds of £20,000/QALY, €20,000/QALY and €30,000/QALY in 79.6%, 75.5% and 97.3% of model runs for the UK, Spain and France, respectively.

**Conclusions:**

Empagliflozin added to SoC leads to health benefits for patients with HFrEF and is a cost-effective treatment option for payers in multiple European countries (UK, Spain, France).

**Supplementary Information:**

The online version contains supplementary material available at 10.1007/s10198-022-01555-6.

## Introduction

Heart failure (HF) is characterised by frequent hospitalisations and significantly diminished life expectancy and health-related quality of life (HRQoL), imposing a significant humanistic and economic burden in Europe and internationally [1]. While recent figures are lacking, HF-related expenditures in the European Union were estimated at €29 billion in 2012, driven largely by recurrent and prolonged hospitalisations [2]. European incidence of HF is approximately 5 per 1000 patient-years (PYs) [3, 4], while recent age-standardised estimates suggest prevalence ranges from 703.8/100,000 people (95% confidence interval [CI]: 609.6, 801.5) in Eastern Europe to 1058.1/100,000 people (95% CI 925.5, 1203.5) in Central Europe [5]. HF disproportionately impacts older individuals, and combined with the projected ageing of the European population, evidence suggests this burden is likely only to grow in the coming years [6].

HF with reduced ejection fraction (HFrEF; i.e. left ventricular ejection fraction of ≤ 40%) comprises approximately half or more of HF cases [4, 7]. As per current European guidelines, the mainstay of treatment for HFrEF is pharmacotherapy, potentially supplemented by device therapy and other interventions which aim to reduce symptoms, improve functional status and HRQoL, and decrease the rate of hospitalisation for worsening heart failure (HHF) [8]. Despite these interventions, prognosis for these patients generally remains poor [7].

Recent large-scale clinical trials (DAPA-HF and EMPEROR-Reduced) have shown the effects of sodium–glucose cotransporter-2 inhibitors (SGLT2i) (dapagliflozin and empagliflozin) on reducing the risk of cardiovascular (CV) death or HHF in patients with HFrEF [9, 10]. EMPEROR-Reduced compared treatment with empagliflozin (Jardiance^®^) vs. placebo, both in addition to background standard of care (SoC), demonstrating a 25% (hazard ratio [HR]: 0.75; 95% CI 0.65, 0.86) reduction in the primary composite outcome of CV death or first HHF and 30% (HR: 0.70; 95% CI 0.58, 0.85) reduction in total HHF [9].

Examining the economic implications of these clinical results is essential to assist decision-makers in making judicious use of scarce healthcare resources, because this facilitates understanding of the trade-offs that ensue when introducing a novel health intervention that may improve clinical outcomes for patients, but may also increase expenditures for local healthcare systems. The establishment of dedicated institutions and frameworks for the assessment of new health technologies in each jurisdiction included in this study attests to the widespread recognition of the importance of formal economic evaluation as a tool for supporting such decisions [11–14]. Accordingly, this research evaluated the cost-effectiveness of adding empagliflozin to SoC compared with SoC alone from the perspective of representative healthcare systems in Europe, namely the United Kingdom (UK), Spain and France.

## Methods

### Model approach

**Fig. 1:**
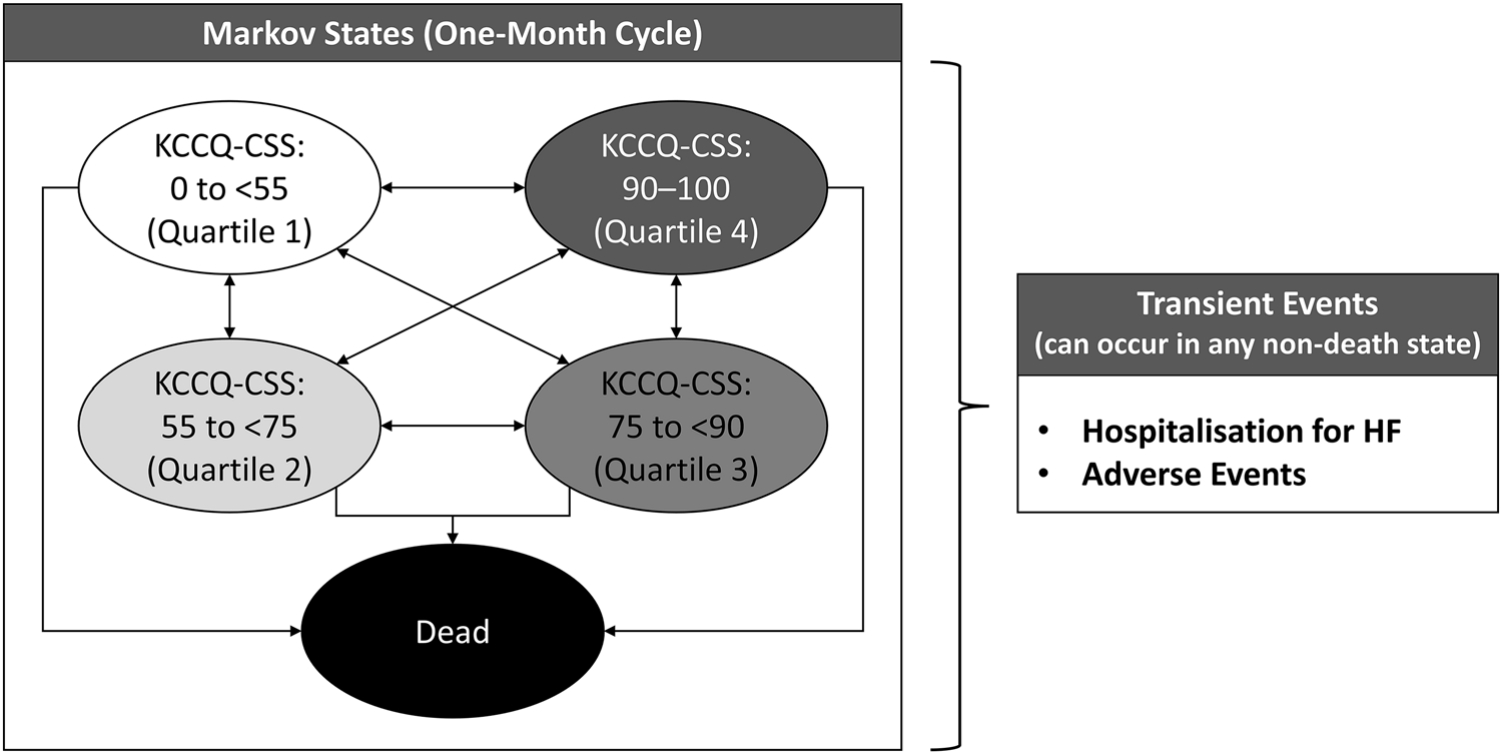
Model diagram. *HF* heart failure, *KCCQ-CSS* Kansas City Cardiomyopathy Questionnaire Clinical Summary Score

A Microsoft Excel^®^-based lifetime Markov cohort model with monthly cycles comparing empagliflozin plus SoC vs. SoC alone was developed to simulate patients’ progression through health states based on the Kansas City Cardiomyopathy Questionnaire (KCCQ) Clinical Summary Score (CSS; Fig. 1), which ranges from 0 to 100, with higher scores indicating better health status. The model consisted of five states that encompassed KCCQ-CSS quartiles and death. Several factors motivated the selection of a Markov cohort approach. Such models employ a simple structure and allow for rapid execution of analyses yet possess sufficient flexibility to account for patient heterogeneity through subgroup analyses and can capture both the short- (e.g. episodes of HHF) and long-term impacts of treatment (e.g. disease progression, as reflected in transitions between KCCQ-CSS quartiles). This approach also aligns with a recent health economic evaluation of dapagliflozin in HFrEF [15].

The KCCQ-CSS was selected as the basis for the health states used in the model because the KCCQ tool is an established and prognostically important patient-reported measure of health status in HFrEF [16–19] that health technology assessment bodies regard as appropriate for decision-making, sidestepping issues associated with alternative measures such as New York Heart Association (NYHA) functional classifications (these issues are summarised in the Discussion section). Of note, the KCCQ-CSS is more comprehensive than the measure employed in the aforementioned dapagliflozin study (i.e. the KCCQ Total Symptom Score [TSS]), encompassing not merely symptom burden and frequency, but also physical limitation.

### Model description

The modelled cohort was partitioned across the four KCCQ-CSS health states at baseline. In each cycle, patients could remain in the same state, progress to a lower (worse) KCCQ-CSS state, regress to a higher (better) KCCQ-CSS state, or die, and could experience HHF or treatment-related adverse events (AEs) as transient events while in a KCCQ-CSS state. The model monitored patients’ HRQoL and resource use over time as they transitioned between health states and independently tracked CV- and non-CV-related deaths. Treatment was assumed to impact patient outcomes by reducing the risk of mortality and HHF and influencing the likelihood of transitions between KCCQ-CSS health states, further modifying the risk of model events and driving accrual of both costs and quality-adjusted life-years (QALYs).

Modelled SoC therapies represented treatments patients in the EMPEROR-Reduced trial received, including diuretics, inhibitors of the renin–angiotensin system and neprilysin, beta-blockers, mineralocorticoid receptor antagonists, and cardiac devices (when indicated). Similar to the trial, where termination of therapy could result from exposure to AEs, non-adherence, or other causes, the model assumed patients for whom SoC was complemented by empagliflozin gradually discontinued empagliflozin therapy over time, after which they received SoC alone until death or the model horizon expired.

The model structure was the same for the UK, Spain and France, although some inputs used to inform each analysis were country-specific (Supplementary Appendix Section S1). Direct medical costs consisted of expenditures related to drug acquisition, clinical event management, and disease management. Utilities were accrued based on time spent in each KCCQ-CSS state, adjusted for disutilities associated with HHF and AEs. Future costs and benefits were discounted at annual rates of 3.5%, 3.0% and 2.5% for the UK [12], Spain [20] and France [21], respectively. The primary model outcome was the incremental cost-effectiveness ratio (ICER), expressed as cost per QALY gained, measuring the additional cost to gain one QALY by treating patients with empagliflozin plus SoC vs. SoC alone.

### Population

The modelled cohort was representative of patients in the EMPEROR-Reduced trial [9]. Patients were initially distributed into KCCQ-CSS quartiles as follows: 0 to < 55 (24.3%), 55 to < 75 (25.1%), 75 to < 90 (27.2%) and 90–100 (23.4%). The mean starting age of the cohort was 67 years and 76% were male. Half the patients had history of type 2 diabetes (T2D), 48% had an estimated glomerular filtration rate (eGFR) less than 60 mL/min/1.73 m^2^, and 20% were receiving an angiotensin receptor–neprilysin inhibitor (ARNi) as part of SoC.

The model was equipped to perform a range of pre-specified subgroup analyses based on patient age at baseline (< 65 vs. ≥ 65 years), presence or absence of T2D, eGFR (< 60 vs. ≥ 60 mL/min/1.73 m^2^) and utilisation or non-utilisation of ARNi.

### Disease and treatment effect

#### Disease progression or regression

EMPEROR-Reduced trial data were used to estimate transition probability matrices to capture improvement (ascent) or progression (descent) of disease through KCCQ-CSS quartiles. KCCQ-CSS quartiles were used to ensure sufficient granularity in predicting movement between health states and were constructed in such a way as to ensure enough patient data for robust statistical analysis (i.e. quartile 1: 0 to < 55; quartile 2: 55 to < 75; quartile 3: 75 to < 90; quartile 4: 90–100). Treatment-specific monthly transition probabilities between quartiles were computed based on transition count data. Independent transition matrices were developed based on the first three months of follow-up in EMPEROR-Reduced, from month 4 to 8, and from month 9 onwards, recognising inflection points observed in the data (Supplementary Appendix Section S3). Transition probabilities were assumed to be equivalent for all patient subgroups.

#### Treatment discontinuation

Parametric survival analysis of EMPEROR-Reduced trial data was conducted to estimate time to empagliflozin treatment discontinuation (following an exponential distribution), with time-varying KCCQ-CSS as covariates (see Supplementary Appendix for statistical methods [Section S2] and results [Section S3]); alternative distributions were considered in sensitivity analyses. Discontinuation from empagliflozin was assumed to result in a treatment effect on event risks, costs, and utilities similar to SoC alone. This is a conservative assumption, to the extent it implies the treatment effect of empagliflozin dissipates immediately upon discontinuation, which may not be true in practice. However, event risks for patients who discontinue empagliflozin will remain lower than for patients who only ever received SoC, due to differences in how patients are distributed across health states. These distributions enter the predictive risk equations for HHF and all-cause and CV death, and differences among them, which persist even after discontinuing treatment with empagliflozin, will result in sustained divergence in risk profiles between interventions.

#### Transient event risks

Risk of first and subsequent HHF was estimated using a Poisson regression model fitted to patient-level EMPEROR-Reduced data with generalised estimating equations to account for the repeated measures on patients (Supplementary Appendix Section S2), including treatment allocation and time-varying KCCQ-CSS health states as covariates (Supplementary Appendix Section S3). Being on treatment with empagliflozin plus SoC or residing in higher KCCQ-CSS quartiles was associated with lower risk of HHF.

The model captured AEs experienced by patients in the EMPEROR-Reduced trial, including urinary tract infection, genital mycotic infection, acute renal failure, hepatic injury, volume depletion, hypotension, hypoglycaemic events and bone fracture. Patients were subject to ongoing risk of experiencing AEs, estimated from the trial data (Supplementary Appendix Section S4). A constant hazard was assumed for each AE.

#### Mortality

Parametric survival analyses were undertaken using the complete EMPEROR-Reduced dataset to capture all-cause and CV death as a function of treatment and time-varying KCCQ-CSS, both during and beyond the trial duration. Distributions chosen for all-cause and CV death (Weibull) were selected from potential distributions based on goodness of fit and clinical plausibility of long-term projections; alternative distributions were considered in sensitivity analyses. Details regarding the statistical methodology and results of the parametric survival analyses are presented in Supplementary Appendices Sections S2 and S3, respectively.

The model independently tracked CV and non-CV deaths; whereas CV death was dictated by the corresponding predictive risk equation (see above), non-CV death was calculated as (a) the difference between parametric fits for all-cause and CV death or (b) the difference between rates of age- and sex-specific all-cause mortality predicted from country-specific life tables [22–24] and rates of CV death recorded in national cause-of-death registries [25–27]—whichever was largest. This was done to ensure the risk of non-CV death was always at least as high as that observed in the population more generally.

### Utilities

The model applied utility scores ranging from 0 (death) to 1 (full health) to capture the effect of disease severity and clinical events on HRQoL. QALYs accrued during each model cycle, calculated as the difference between KCCQ-CSS health state utilities, and utility decrements attributable to transient events, including HHF and AEs (Table 1; also see Supplementary Appendix Section S5). The impact of transient events on HRQoL was captured as one-off decrements to the proportion of patients experiencing the event and assumed to apply during the month in which those events occurred (i.e. for one model cycle).

**Table 1:** Summary of key base-case input parameters

Input parameter	UK	Spain	France	Sources
Discount rate: cost	3.5%	3.0%	2.5%	[12, 20, 21]
Discount rate: health	3.5%	3.0%	2.5%	[12, 20, 21]
Distribution for clinical parameters				
HHF	Poisson	Poisson	Poisson	EMPEROR-Reduced
Time to CV death	Weibull	Weibull	Weibull	EMPEROR-Reduced
Time to all-cause death	Weibull	Weibull	Weibull	EMPEROR-Reduced
Time to empagliflozin treatment discontinuation	Exponential	Exponential	Exponential	EMPEROR-Reduced
Utility parameters				
Health state utility: KCCQ-CSS 0 to < 55 (Q1)	0.520	0.629	0.464	EMPEROR-Reduced
Health state utility: KCCQ-CSS 55 to < 75 (Q2)	0.637	0.754	0.610	EMPEROR-Reduced
Health state utility: KCCQ-CSS 75 to < 90 (Q3)	0.710	0.832	0.721	EMPEROR-Reduced
Health state utility: KCCQ-CSS 90–﻿100 (Q4)	0.774	0.891	0.810	EMPEROR-Reduced
Disutility: HHF	– 0.246	– 0.291	– 0.240	EMPEROR-Reduced
Disutility: urinary tract infection	– 0.025	– 0.025	– 0.025	[30]
Disutility: genital mycotic infection	– 0.058	– 0.053	– 0.052	EMPEROR-Reduced
Disutility: acute renal failure	– 0.010	– 0.014	– 0.011	EMPEROR-Reduced
Disutility: hepatic injury	– 0.016	– 0.011	– 0.018	EMPEROR-Reduced
Disutility: volume depletion	– 0.018	– 0.015	– 0.017	EMPEROR-Reduced
Disutility: hypotension	– 0.025	– 0.025	– 0.025	[29]
Disutility: hypoglycaemic event	– 0.048	– 0.041	– 0.055	EMPEROR-Reduced
Disutility: bone fracture	– 0.165	– 0.170	– 0.148	EMPEROR-Reduced
Cost parameters				
Monthly drug acquisition cost: empagliflozin + SoC	£79	€157	€129	[39–41]
Monthly drug acquisition cost: SoC	£40	€100	€84	[39–41]
Event management cost: HHF	£3072	€3814	€4968	[42–44]
Event management cost: CV death	£4146	€6276	€3764	[42, 45, 46]
Event management cost: urinary tract infection	£40	€57	€36	[43, 47–53]
Event management cost: genital mycotic infection	£40	€57	€36	[43, 47–53]
Event management cost: acute renal failure	£1906	€4243	€3739	[42, 44, 54]
Event management cost: hepatic injury	£1274	€2709	€1055	[42–44, 46–53]
Event management cost: volume depletion	£40	€57	€36	[43, 47–53]
Event management cost: hypotension	£40	€57	€36	[43, 47–53]
Event management cost: hypoglycaemic event	£627	€1461	€2319	[42–44, 47–53, 55]
Event management cost: bone fracture	£2710	€5042	€3352	[42, 44, 46]
Disease management cost: KCCQ-CSS 0 to < 55 (Q1)	£77	€55	€48	[43, 44, 47–53, 56–59]
Disease management cost: KCCQ-CSS 55 to < 75 (Q2)	£77	€55	€48	[30, 43, 44, 47–53, 56–59]
Disease management cost: KCCQ-CSS 75 to < 90 (Q3)	£77	€55	€48	[30, 43, 44, 47–53, 56–59]
Disease management cost: KCCQ-CSS 90–100 (Q4)	£77	€55	€48	[30, 43, 44, 47–53, 56–59]

*CV* cardiovascular, *HHF* hospitalisation for worsening heart failure, *KCCQ-CSS* Kansas City Cardiomyopathy Questionnaire Clinical Symptom Score, *SoC* standard of care, *UK* United Kingdom

EQ-5D-5L responses collected in EMPEROR-Reduced were mapped to EQ-5D-3L using crosswalks formulated by van Hout et al. [28], which were then converted to index scores using published value sets for each of the three countries. Linear mixed-effects regression models (see Supplementary Appendix Section S3) were constructed to predict utility values from baseline demographics and medical history, time-varying KCCQ-CSS and clinical events (HHF, AEs).

Utility decrements attributable to HHF were estimated as a weighted average of the relevant coefficients from the regression models, each representing a different duration of time elapsed since the event occurred, and those durations themselves. Disutility estimates for most AEs were sourced directly from the regression model; however, urinary tract infection and hypotension in EMPEROR-Reduced could not be derived from the trial analysis due to low incidence and were obtained from published literature [29, 30].

An age adjustment was applied to KCCQ-CSS health state utility values to account for differences between the EMPEROR-Reduced trial and country-specific population norms; in particular, the model assumed HRQoL in KCCQ-CSS quartile 4 would resemble the population more generally, thereby ensuring utilities for patients in lower quartiles (i.e. whose conditions were more severe) would not be higher than this level. The model did not apply further adjustments to account for cohort ageing due to comparatively brief life expectancy for patients with HFrEF.

### Costs and resource utilisation

Direct reimbursable medical care costs were expressed in appropriate national currencies (i.e. 2021 British pounds for the UK and 2021 Euros for Spain and France), with adjustments for inflation. The model included costs associated with drug acquisition, management of clinical events, and disease management, sourced from local costing databases and published literature, as appropriate (Table 1). Detailed itemisation of costs incorporated into the model is supplied in Supplementary Appendix Section S6.

Drug costs for empagliflozin 10 mg and SoC therapies were extracted from national databases in the three country settings—Monthly Index of Medical Specialities for the UK, BotPlus Web Database in Spain (retail plus value added tax for the base-case analysis and ex-factory price for sensitivity analysis) and the Official Journal in France (public prices including all taxes). For each SoC HF medication class, costs of individual therapies were used to compute an average cost, assuming treatments within each class were uniformly distributed (UK and Spain) or based on local market share (France). Then, the drug cost of SoC was computed as a weighted average cost based on these values and utilisation of HF medication classes at baseline in the empagliflozin plus SoC and SoC arms of EMPEROR-Reduced. Finally, a cost associated with each treatment regimen (i.e. empagliflozin plus SoC and SoC alone) was computed. Indicated strength and dosage for each drug were based on summaries of product characteristics. The cost of empagliflozin 10 mg was incurred until treatment discontinuation, whereas expenditures associated with SoC accrued until death or the model horizon expired. Detailed examples of the steps involved in the calculation of treatment costs are presented in Supplementary Appendix Section S6.

The model assumed that patients receiving implantable cardioverter defibrillators and cardiac resynchronisation therapy had these devices installed prior to entry, and, accordingly, the analysis excludes expenditures associated with the devices or their implantation. In addition, it was assumed patients entering the model were already receiving appropriately titrated doses of SoC therapies, such that the stable maintenance dosage for each SoC treatment could be applied.

Expenses for managing clinical events (HHF, CV death and AEs) were obtained from national databases or published literature and modelled as one-off costs. The model assumed no cost for non-CV death. For AEs, these were calculated with reference to the distribution of visit types (i.e. outpatient or inpatient), reflecting their typical severity, and corresponding costs.

HF-related disease management expenditures accrued each cycle and were assumed similar across KCCQ-CSS quartiles, reflecting utilisation rates and local costs associated with primary care, cardiologist visits, and accident and emergency referrals.

### Model validation

Validation steps included assessment of face validity, technical validity, predictive validity, and cross-validity. Thorough evaluation of face validity and technical validity was supported by an independent modelling expert using the TECHnical VERification checklist (TECH-VER) [31]. Meanwhile, predictive validity was assessed by comparing model-predicted rates of HHF, CV and all-cause death against observed rates from EMPEROR-Reduced (Supplementary Appendix Section S7), while cross-validity entailed identifying and explicating differences in analytical results relative to a recently published model with a similar decision problem [15].

To assess the applicability and generalisability of this study to clinical practice in each jurisdiction, baseline characteristics and observed event rates for EMPEROR-Reduced participants were compared with data collected from recent real-world studies involving patients with HFrEF, including Incidence, Prevalence, and resoUrce utiLiSation of hEart failure in England (PULSE), the FREnch Survey on Heart Failure (FRESH), and published analyses undertaken using a large Spanish administrative database [32, 33]; the assessment suggested this analysis may generalise to clinical practice in the UK, France and Spain more broadly, although this should be interpreted cautiously due to the limitations of the available real-world evidence (RWE). Overviews of the key features and limitations of each study and comparisons of patient baseline characteristics and clinical outcomes are reported in Supplementary Appendix Section S7.

### Analyses

The base-case analysis predicted absolute and incremental lifetime cumulative events per 100 PYs, life-years (LY), QALYs and costs with empagliflozin plus SoC vs. SoC alone for the EMPEROR-Reduced trial intent-to-treat (ITT) population. Additionally, the model synthesised results for incremental costs and QALYs to compute the ICER for empagliflozin plus SoC vs. SoC alone. The ICERs for each setting were compared with country-specific, willingness-to-pay (WTP) thresholds—£20,000/QALY for the UK [12], €20,000/QALY for Spain [34], and €30,000/QALY for France [35].

Scenarios were run for pre-specified subgroups that were considered clinically and/or economically meaningful (outlined above). Deterministic sensitivity analyses (DSA) were performed using plausible ranges or alternative values for key inputs (i.e. model horizon and discount rates; KCCQ-CSS utilities and utility decrements for AEs and HHF; costs associated with drug acquisition and clinical event/disease management; distributions and treatment effects for clinical outcomes; and whether to exclude treatment discontinuation or the life table adjustment for non-CV mortality) to test the robustness of model results to changes in the parameters. A probabilistic sensitivity analysis (PSA) was conducted using distributions reflecting parameter uncertainties, producing 1000 pairs of incremental effectiveness and cost estimates. The parameters for sensitivity analyses are provided in Supplementary Appendix Sections S9 and S10.

## Results

### Base-case analysis

Empagliflozin plus SoC was associated with fewer HHFs, CV deaths, non-CV deaths and specific AEs (acute renal failure, hepatic injury and hypoglycaemic events) vs. SoC alone in all three country settings over a lifetime horizon (Table 2; a detailed breakdown by KCCQ-CSS quartile is also provided in Supplementary Appendix Section S8), consistent with the EMPEROR-Reduced trial results. Due to lower rates of CV and non-CV death, reduced incidence of HHF and improved health status (as reflected in the proportion of time spent in each KCCQ-CSS quartile), patients treated with empagliflozin plus SoC accumulated more discounted LYs (0.18, 0.19 and 0.20 in the UK, Spain and France, respectively), and a larger number of discounted QALYs gained (0.19, 0.23 and 0.21, respectively). Differences in health outcomes reflect the unique demographics of each nation (i.e. variations in background mortality, as captured in life tables, as well as differences in valuation of EQ-5D health states and population norms), local health technology assessment guidelines (i.e. as reflected in discount rates applied to cost and health outcomes), and variation in the finance and delivery of healthcare services across jurisdictions (i.e. as reflected in disparities in healthcare resource utilisation and unit costs).

**Table 2:** Base-case results estimated over lifetime horizon

	UK (GBP)			Spain (EURO)			France (EURO)		
	Empagliflozin + SoC	SoC	Incremental	Empagliflozin + SoC	SoC	Incremental	Empagliflozin + SoC	SoC	Incremental
Clinical events, per 100 PY									
HHF	17.60	20.80	– 3.20	17.60	20.79	– 3.19	17.60	20.79	– 3.19
CV death	9.88	10.34	– 0.46	9.88	10.34	– 0.46	9.89	10.34	– 0.46
Non-CV death	4.41	4.46	– 0.05	4.36	4.42	– 0.05	4.36	4.42	– 0.06
Urinary tract infection	3.94	3.76	0.18	3.94	3.76	0.18	3.94	3.76	0.18
Genital mycotic infection	0.95	0.53	0.42	0.95	0.53	0.42	0.95	0.53	0.42
Acute renal failure	8.58	9.02	– 0.44	8.58	9.02	– 0.44	8.58	9.02	– 0.44
Hepatic injury	3.63	3.83	– 0.20	3.63	3.83	– 0.20	3.63	3.83	– 0.20
Volume depletion	9.01	8.76	0.25	9.01	8.76	0.25	9.01	8.76	0.25
Hypotension	7.95	7.69	0.26	7.95	7.69	0.26	7.95	7.69	0.26
Hypoglycaemic event	1.23	1.25	– 0.02	1.23	1.25	– 0.02	1.23	1.25	– 0.02
Bone fracture	1.95	1.89	0.06	1.95	1.89	0.06	1.95	1.89	0.06
Total cost	£16,661	£15,475	£1185	€24,319	€22,549	€1770	€21,726	€20,542	€1183
Treatment costs	£4240	£2673	£1567	€9304	€6950	€2353	€7895	€5947	€1947
HHF and CV death management costs	£5418	£5964	– £546	€7569	€8261	– €692	€7529	€8392	– €863
AE management costs	£1615	£1620	– £5	€3513	€3529	– €16	€2810	€2824	– €14
Disease management costs	£5388	£5219	£169	€3933	€3808	€125	€3492	€3379	€113
Total LYs	5.81	5.62	0.18	5.96	5.77	0.19	6.11	5.91	0.20
Total QALYs	3.76	3.57	0.19	4.51	4.28	0.23	3.98	3.77	0.21
ICER	–	–	£6152	–	–	€7736	–	–	€5511

*AE* adverse event, *CV* cardiovascular, *GBP* Great British pound, *HHF* hospitalisation for worsening heart failure, *ICER* incremental cost-effectiveness ratio, *LY* life-years, *PY* patient-years, *QALY* quality-adjusted life-year, *SoC* standard of care, *UK* United Kingdom

As shown in Table 2, empagliflozin plus SoC vs. SoC alone led to additional (discounted) lifetime costs in the UK (£1185/patient), Spain (€1770/patient) and France (€1183/patient), due primarily to higher drug acquisition but partially also to increased disease management costs, which were somewhat offset by savings from clinical events avoided (primarily reduced HHF). Relative to SoC, empagliflozin plus SoC yielded ICERs of £6152/QALY, €7736/QALY and €5511/QALY in the UK, Spain and France, respectively. Considering local WTP thresholds of £20,000/QALY for the UK, €20,000/QALY for Spain and €30,000/QALY for France, these results indicate that empagliflozin plus SoC is cost-effective compared to SoC alone for treatment of patients with HFrEF in all three settings.

### Sensitivity and subgroup analyses

**Fig. 2:**
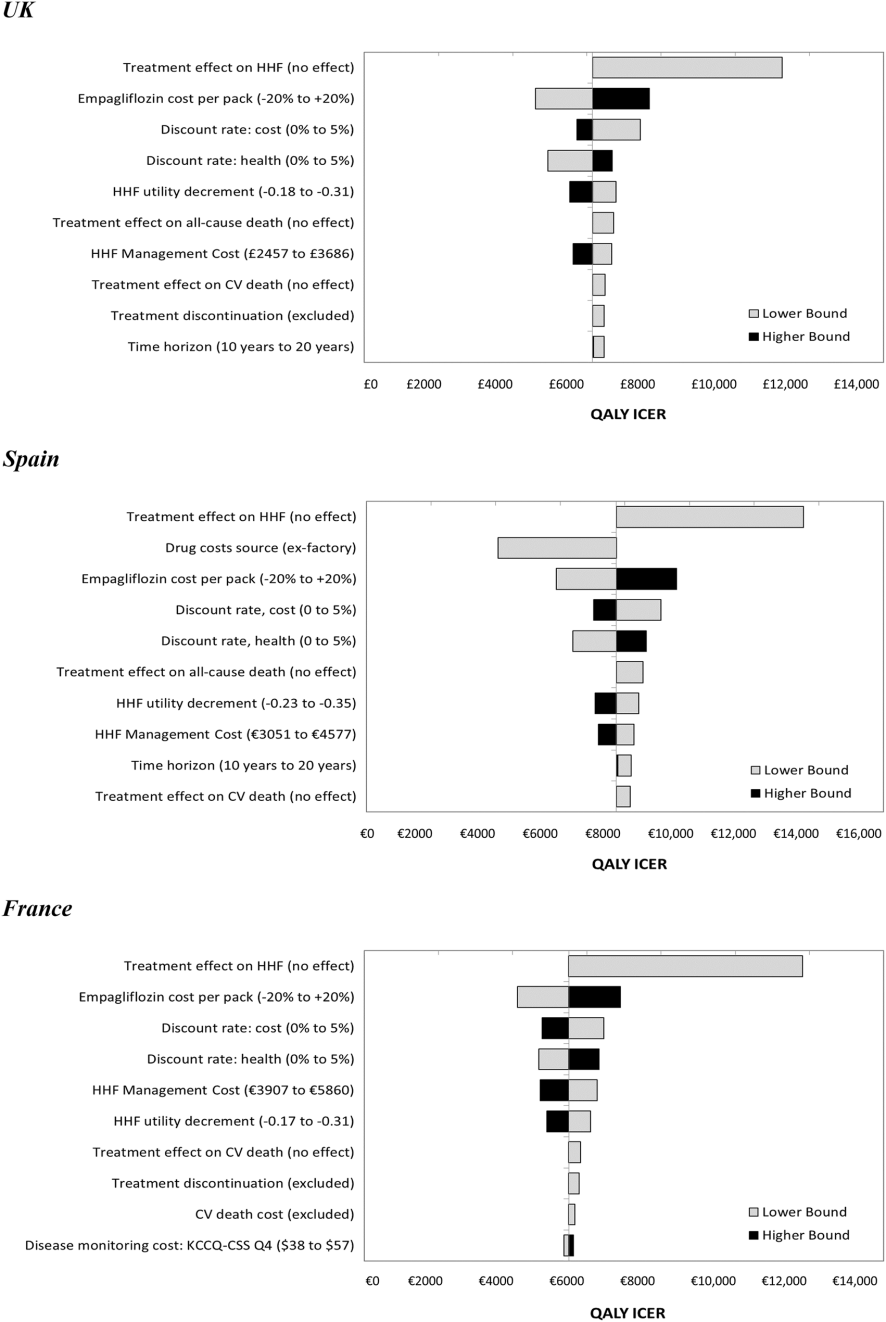
Deterministic sensitivity analyses (top 10 scenarios). *CV* cardiovascular, *HHF* hospitalisation for worsening heart failure, *ICER* incremental cost-effectiveness ratio, *KCCQ-CSS* Kansas City Cardiomyopathy Questionnaire Clinical Summary Score, *QALY* quality-adjusted life-year, *UK* United Kingdom

Empagliflozin plus SoC remained cost-effective compared with SoC alone in DSA considering local WTP thresholds from all three payer perspectives, with ICERs ranging from £4615–£11,258 in the UK (< £20,000/QALY), €4073–€13,520 in Spain (< €20,000/QALY) and from €4123–€11,810 in France (< €30,000/QALY), reinforcing the base-case results. Eliminating the effect of treatment on HHF was the most influential parameter, increasing the ICER in each jurisdiction. That was followed by empagliflozin cost per pack (− 20 to + 20%) and discount rate for cost (0–5%, vs. 3.5% in the base-case) in the UK; drug costing source (ex-factory, vs. retail in the base-case) and empagliflozin cost per pack (− 20 to + 20%) in Spain; and empagliflozin cost per pack (− 20 to + 20%) and discount rate for cost (0–5%, vs. 2.5% in the base-case) in France (Fig. 2; also see Supplementary Appendix Section S9).

**Fig. 3:**
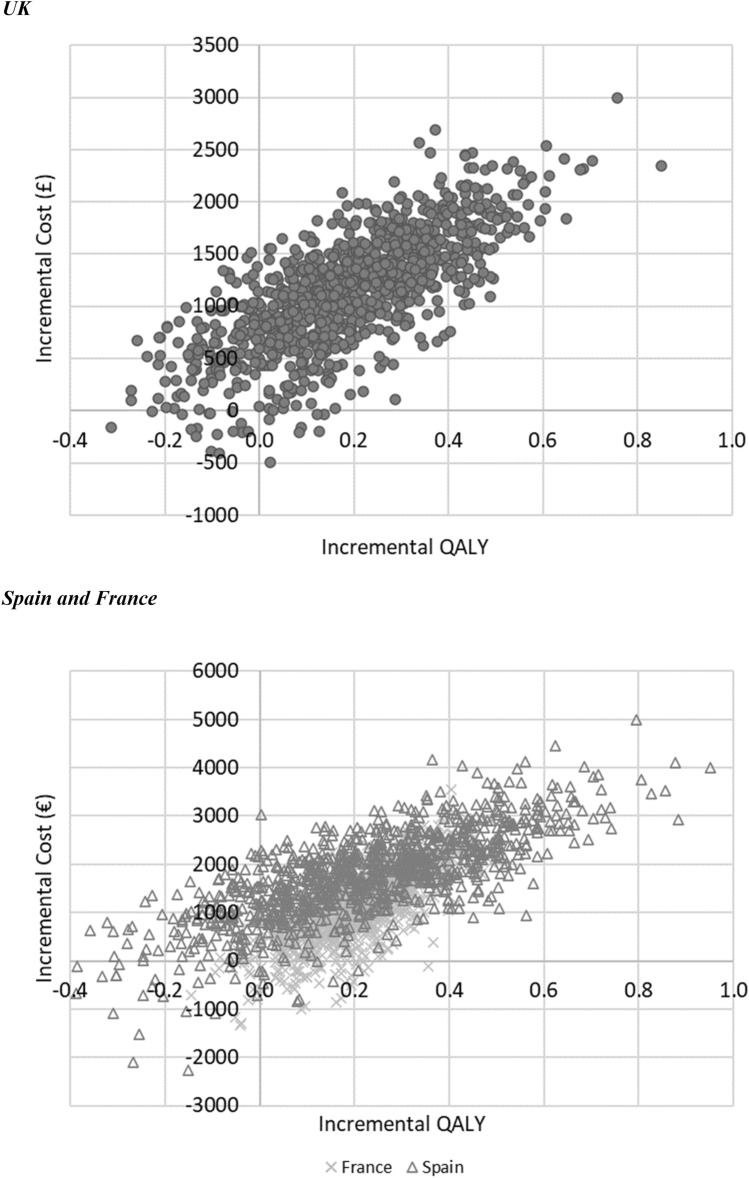
Incremental cost-effectiveness scatterplots. *QALY* quality-adjusted life-year, *UK* United Kingdom

The PSA results found that the mean ICERs for the UK, Spain and France were £6061/QALY, €7788/QALY and €5409/QALY, respectively. The chance of empagliflozin plus SoC vs. SoC being cost-effective at WTP thresholds of £20,000/QALY (UK), €20,000/QALY (Spain) and €30,000/QALY (France) were 79.6%, 75.5% and 97.3%, respectively (Fig. 3; also see Supplementary Appendix Section S10).

The cost-effectiveness of empagliflozin plus SoC vs. SoC alone was preserved across all subgroups considered in the analysis for each jurisdiction (Table 3). The model predicted empagliflozin plus SoC to be most cost-effective in the subgroup of patients aged < 65 years (UK: ICER of £3702/QALY; Spain: ICER of €5033/QALY; and France: ICER of €2256/QALY), followed by patients with T2D (UK: ICER of £4899/QALY; Spain: ICER of €6405/QALY; and France: ICER of €3501/QALY).

**Table 3:** Results of subgroup analyses

	ICER-QALY (percent change in ICER from base case)		
Subgroup	UK	Spain	France
No T2D	£6996 (14%)	€8533 (10%)	€6959 (26%)
T2D	£4899 (– 20%)	€6405 (– 17%)	€3501 (– 36%)
Age < 65 years	£3702 (– 40%)	€5033 (– 35%)	€2256 (– 59%)
Age ≥ 65 years	£7571 (23%)	€9205 (19%)	€7467 (35%)
eGFR < 60 mL/min/1.73 m^2^	£6689 (9%)	€8174 (6%)	€6806 (24%)
eGFR ≥ 60 mL/min/1.73 m^2^	£5581 (– 9%)	€7323 (– 5%)	€3887 (– 29%)
Treated with ARNi	£6305 (2%)	€8501 (10%)	€7448 (35%)
Not treated with ARNi	£6681 (9%)	€8356 (8%)	€5480 (– 1%)

*ARNi* angiotensin receptor–neprilysin inhibitor, *eGFR* estimated glomerular filtration rate, *ICER* incremental cost-effectiveness ratio, *QALY* quality-adjusted life-year, *T2D* type 2 diabetes, *UK* United Kingdom

## Discussion

A lifetime Markov cohort model was developed to assess the cost-effectiveness of empagliflozin plus SoC compared with SoC alone for the treatment of adults with HFrEF in the UK, Spain and France, drawing upon patient-level data from the EMPEROR-Reduced trial.

Base-case results demonstrate that supplementing SoC with empagliflozin generated clinical benefit for patients via three distinct but connected channels. First, empagliflozin plus SoC was associated with a significant treatment effect in the predictive risk equation for HHF, a transient event that significantly detracts from HRQoL. Second, similar treatment effects in the risk equations for all-cause and CV death culminated in increased average life and quality-adjusted life expectancy. Finally, treatment influenced transitions between KCCQ-CSS quartiles, and, by extension, the distribution of the cohort across quartiles over time; this directly contributed to patient HRQoL (because quartiles associated with higher KCCQ-CSS are also associated with higher utilities) and drove further differences in the risk of HHF and mortality. Operating through these channels, empagliflozin plus SoC resulted in lower rates of HHF or CV death compared with SoC alone, culminating in additional LYs and higher QALYs. Utilising empagliflozin alongside SoC also produced higher monthly drug acquisition costs, and greater life expectancy vs. SoC which further increased lifetime expenditures for medication, as well as for disease management. These were partially offset by cost-savings from reduced incidence of clinical events, especially HHF. ICERs capturing the trade-offs between increased clinical benefits and higher lifetime costs fall well below accepted WTP thresholds in each jurisdiction, demonstrating that empagliflozin plus SoC was highly cost-effective compared with SoC alone. These findings were broadly supported by results from a series of comprehensive scenario and sensitivity analyses.

The modelling approach adopted in this analysis was consistent with a recently published economic evaluation in HFrEF for the SGLT2i dapagliflozin added to SoC [15]. This model, however, used the KCCQ-CSS instead of the KCCQ-TSS applied in McEwan et al., 2020. The KCCQ-CSS is a clinically more comprehensive measure than KCCQ-TSS, since it accounts for both the TSS domains (symptom frequency and burden) and physical limitations. Cross-validation of model predictions vs. the dapagliflozin model found higher rates of HHF and CV death in the former. This is as anticipated, given that EMPEROR-Reduced was enriched for higher-risk patients (e.g. reduced ejection fraction and higher levels of natriuretic peptides) compared to DAPA-HF [9].

This model has several strengths. First, it accurately reproduced the EMPEROR-Reduced trial results over the median trial follow-up period of 16 months. Second, health status was quantified using the KCCQ-CSS (continuously scaled from 0 to 100 to assess status from very poor to excellent), a prognostically important, patient-reported measure of health status which is considered more reliable than physician-reported NYHA classification [36]. KCCQ-CSS better captured improvement or progression of disease via transitions of patients between KCCQ-CSS-based health states compared with NYHA classification. Exploratory analyses of EMPEROR-Reduced trial data showed little movement in NYHA health state occupancy (i.e. patients tended to remain in class II/III) over the trial duration. Lastly, non-CV death rates were adjusted using local life tables.

The model was also subject to limitations. First, EMPEROR-Reduced collected relatively short-term data, as is typical for a randomised controlled trial. Accordingly, long-term outcomes based on this trial data were extrapolated and are therefore prone to uncertainty. However, sensitivity analyses results imply that the choice of parametric distributions for key clinical outcomes did not significantly impact the estimated ICER, suggesting this residual uncertainty is unlikely to alter the conclusions of the analysis.

Second, as is common in health economic assessments, it is challenging to ascertain the generalisability of the study results to clinical practice in each country considered in the analysis. While a review of real-world studies suggests the baseline characteristics of participants in EMPEROR-Reduced and trial outcomes are reasonably representative of the attributes of patients with HF and outcomes associated with delivery of SoC in each jurisdiction, such comparisons are hindered by significant limitations in the available RWE, including small sample sizes and high proportions of missing data. Furthermore, the inclusion of relatively few participants from any individual country in the EMPEROR-Reduced trial precluded the estimation of country-specific clinical inputs and necessitated the assumption that these inputs are similar across jurisdictions, mirroring challenges identified in another recent health economic evaluation of SGLT2i for the treatment of HFrEF [15]. However, the finding that the cost-effectiveness of empagliflozin plus SoC vs. SoC alone is preserved across subgroups and irrespective of uncertainty in any individual model parameter or structural assumption strongly suggests the central findings of this analysis are robust to country-specific variability in patient characteristics or clinical inputs. This said, ongoing data generation to establish the cost-effectiveness of empagliflozin in clinical practice remains an important objective for future research.

Third, due to inclusion criteria applied to the design of EMPEROR-Reduced, estimated utilities for patients occupying the highest KCCQ-CSS quartile (i.e. Quartile 4) were relatively high considering a population consisting of patients living with HFrEF. While the model applied an adjustment to ensure utility weights for KCCQ-CSS Quartile 4 did not exceed population norms in each country, the adjusted weights might still not be fully representative of this subset of patients. This said, utility weights assigned to the KCCQ-CSS quartiles were not significant drivers of model results in sensitivity analyses, and, accordingly, the cost-effectiveness of empagliflozin plus SoC should be robust to uncertainty around these values.

Fourth, the model did not include diabetic ketoacidosis, a rare complication of SGLT2is. Very few cases of diabetic ketoacidosis were observed in the EMPEROR-Reduced trial, aligned with other results for empagliflozin from the EMPEROR-Preserved trial [37] in HF with preserved ejection fraction and the EMPA-REG OUTCOME trial in patients with T2D and established CV disease [38]. In all trials, very few cases were recorded and no imbalance existed between treatment groups.

In conclusion, the results of this economic evaluation based on extrapolation of EMPEROR-Reduced trial data demonstrated that compared to SoC alone, empagliflozin added to SoC generates incremental health benefits and represents a cost-effective use of healthcare resources for managing patients with HFrEF from the perspective of payers in the UK, Spain and France.

### Supplementary Information

Below is the link to the electronic supplementary material.

Supplementary file1 (DOCX 231 KB)

## Data Availability

All data used in this study are informed based on published literature, national costing databases, or patient-level analysis of the EMPEROR-Reduced trial. Our study data (which is based on de‐ identified data from a clinical trial) is not in a repository, but is available upon reasonable request from the corresponding author.

## References

[1] Braunschweig F, Cowie MR, Auricchio A (2011). What are the costs of heart failure?. Europace.

[2] Cook C, Cole G, Asaria P, Jabbour R, Francis DP (2014). The annual global economic burden of heart failure. Int J Cardiol..

[3] Meyer S, Brouwers FP, Voors AA (2015). Sex differences in new-onset heart failure. Clin Res Cardiol..

[4] Brouwers FP, de Boer RA, van der Harst P (2013). Incidence and epidemiology of new onset heart failure with preserved vs. reduced ejection fraction in a community-based cohort: 11-year follow-up of PREVEND. Eur Heart J..

[5] Bragazzi NL, Zhong W, Shu J (2021). Burden of heart failure and underlying causes in 195 countries and territories from 1990 to 2017. Eur. J. Prev. Cardiol..

[6] The Heart Failure Policy Network. Heart failure policy and practice in Europe. https://www.hfpolicynetwork.org/wp-content/uploads/Heart-failure-policy-and-practice-in-Europe.pdf. Accessed 27 Oct 2021

[7] Murphy SP, Ibrahim NE, Januzzi JL (2020). Heart failure with reduced ejection fraction: a review. JAMA.

[8] European Society of Cardiology. ESC Guidelines for the diagnosis and treatment of acute and chronic heart failure. https://www.escardio.org/Guidelines/Clinical-Practice-Guidelines/Acute-and-Chronic-Heart-Failure. Accessed 1 Sep 2021

[9] Packer M, Anker SD, Butler J (2020). Cardiovascular and renal outcomes with empagliflozin in heart failure. N. Engl. J. Med..

[10] McMurray JJV, Solomon SD, Inzucchi SE (2019). Dapagliflozin in patients with heart failure and reduced ejection fraction. N. Engl. J. Med..

[11] Haute Autorité de santé. Doctrine of the Commission for Economic and Public Health Evaluation CEESP evaluation principles for healthcare products for pricing purposes. https://www.has-sante.fr/upload/docs/application/pdf/2021-09/doctrine_de_la_ceesp_version_anglaise_2021-09-29_11-14-2_803.pdf. Accessed 12 July 2022

[12] National Institute for Health and Care Excellence. Guide to the methods of technology appraisal 2013. https://www.nice.org.uk/process/pmg9/resources/guide-to-the-methods-of-technology-appraisal-2013-pdf-2007975843781. Accessed 15 Oct 2021 27905712

[13] CatSalut. GUÍA Y RECOMENDACIONES PARA LA REALIZACIÓN Y PRESENTACIÓN DE EVALUACIONES ECONÓMICAS Y ANÁLISIS DE IMPACTO PRESUPUESTARIO DE MEDICAMENTOS EN EL ÁMBITO DEL CATSALUT. https://catsalut.gencat.cat/web/.content/minisite/catsalut/proveidors_professionals/medicaments_farmacia/farmaeconomica/caeip/gaeip_publica_castellano_octubre2014_catsalut.pdf. Accessed 26 July 2022

[14] Lopez-Bastida J, Oliva J, Antonanzas F (2010). Spanish recommendations on economic evaluation of health technologies. Eur. J. Health Econ..

[15] McEwan P, Darlington O, McMurray JJV (2020). Cost-effectiveness of dapagliflozin as a treatment for heart failure with reduced ejection fraction: a multinational health-economic analysis of DAPA-HF. Eur. J. Heart Fail..

[16] Green CP, Porter CB, Bresnahan DR, Spertus JA (2000). Development and evaluation of the Kansas City Cardiomyopathy Questionnaire: a new health status measure for heart failure. J. Am. Coll. Cardiol..

[17] Myers J, Zaheer N, Quaglietti S, Madhavan R, Froelicher V, Heidenreich P (2006). Association of functional and health status measures in heart failure. J. Card. Fail..

[18] Parissis JT, Nikolaou M, Farmakis D (2009). Self-assessment of health status is associated with inflammatory activation and predicts long-term outcomes in chronic heart failure. Eur. J. Heart Fail..

[19] Sullivan MD, Levy WC, Russo JE, Crane B, Spertus JA (2007). Summary health status measures in advanced heart failure: relationship to clinical variables and outcome. J. Card. Fail..

[20] Attema AE, Brouwer WBF, Claxton K (2018). Discounting in economic evaluations. Pharmacoeconomics.

[21] Haute Autorité de Santé. Choices in methods for economic evaluation. https://www.has-sante.fr/jcms/r_1499251/en/choices-in-methods-for-economic-evaluation. Accessed 1 Sep 2021

[22] Office for National Statistics. National life tables: UK. https://www.ons.gov.uk/peoplepopulationandcommunity/birthsdeathsandmarriages/lifeexpectancies/datasets/nationallifetablesunitedkingdomreferencetables. Accessed 1 Sep 2021

[23] Institut National de la Santé et de la Recherche Médicale (INSERM). Causes Médicales de Décès 2021. https://cepidc.inserm.fr/causes-medicales-de-deces/interroger-les-donnees-de-mortalite. Accessed 29 June 2021

[24] Instituto Nacional de Estadística. Tablas de mortalidad por año, sexo, edad y funciones (Unidades: %1000). https://www.ine.es/jaxiT3/dlgExport.htm?t=27153&nocab=1. Accessed 29 June 2021

[25] Office for National Statistics. Death registrations summary tables - England and Wales. https://www.ons.gov.uk/peoplepopulationandcommunity/birthsdeathsandmarriages/deaths/datasets/deathregistrationssummarytablesenglandandwalesreferencetables. Accessed 1 Sep 2021

[26] Institut National d’Etudes Démographiques. TABLEAU 68 - TABLE DE MORTALITÉ DES ANNÉES 2017 - 2019, données provisoires arrêtées à fin décembre 2020. https://www.ined.fr/fichier/s_rubrique/193/fe_t68_2019.en.xlsx. Accessed 29 June 2021

[27] Instituto Nacional de Estadística. Death according to Cause of Death 2018: Deaths by causes (detailed list), sex and age (National Results; Units: Deaths). https://www.ine.es/jaxi/dlgExport.htm?path=/t15/p417/a2018/l1//&file=01000.px&nocab=1&L=1. Accessed 29 June 2021

[28] van Hout B, Janssen MF, Feng YS (2012). Interim scoring for the EQ-5D-5L: mapping the EQ-5D-5L to EQ-5D-3L value sets. Value Health.

[29] Sullivan PW, Ghushchyan V (2006). Preference-Based EQ-5D index scores for chronic conditions in the United States. Med. Decis. Making.

[30] Sullivan PW, Ghushchyan VH (2016). EQ-5D scores for diabetes-related comorbidities. Value Health..

[31] Buyukkaramikli NC, Rutten-van Molken M, Severens JL, Al M (2019). TECH-VER: a verification checklist to reduce errors in models and improve their credibility. Pharmacoeconomics.

[32] Sicras-Mainar A, Sicras-Navarro A, Palacios B, Varela L, Delgado JF (2022). Epidemiology and treatment of heart failure in Spain: the HF-PATHWAYS study. Rev. Esp. Cardiol. (Engl Ed)..

[33] Escobar C, Varela L, Palacios B (2022). Clinical characteristics, management, and one-year risk of complications among patients with heart failure with and without type 2 diabetes in Spain. Rev. Clin. Esp. (Barc)..

[34] Vallejo-Torres L, Garcia-Lorenzo B, Serrano-Aguilar P (2018). Estimating a cost-effectiveness threshold for the Spanish NHS. Health Econ..

[35] Lizee T, Basch E, Tremolieres P (2019). Cost-effectiveness of web-based patient-reported outcome surveillance in patients with lung cancer. J. Thorac. Oncol..

[36] National Institute for Health and Care Excellence (NICE). Dapagliflozin for treating chronic heart failure with reduced ejection fraction: Technology appraisal guidance. https://www.nice.org.uk/guidance/ta679/resources/dapagliflozin-for-treating-chronic-heart-failure-with-reduced-ejection-fraction-pdf-82609327985605. Accessed 16 July 2022 40737468

[37] Anker SD, Butler J, Filippatos G (2021). Empagliflozin in heart failure with a preserved ejection fraction. N. Engl. J. Med..

[38] Zinman B, Wanner C, Lachin JM (2015). Empagliflozin, cardiovascular outcomes, and mortality in type 2 diabetes. N. Engl. J. Med..

[39] Monthly Index of Medical Specialities. Drug Database. https://www.mims.co.uk/. Accessed 1 Sep 2021

[40] Consejo General de Colegios Farmaceuticos. BOT PLUS web. https://botplusweb.portalfarma.com/botplus.aspx. Accessed 1 July 2021

[41] Legifrance. Avis relatif à l'avenant n° 20 à la convention nationale du 4 avril 2012 organisant les rapports entre les pharmaciens titulaires d'officine et l'assurance maladie. https://www.legifrance.gouv.fr/jo_pdf.do?id=JORFTEXT000041931400. Accessed 1 Sep 2021

[42] Ministerio de Sanidad Consumo y Bienestar Social. Registro de altas - Categoría CIE-10 – CMBD. https://pestadistico.inteligenciadegestion.mscbs.es/. Accessed 12 Feb 2021

[43] Haute Autorité de Santé. Entresto efficiency report. https://www.has-sante.fr/upload/docs/application/pdf/2017-12/entresto_12042016_avis_efficience.pdf. Accessed 19 Aug 2021

[44] National Health Service. National Schedule of NHS Costs-Year 2018–19. https://www.england.nhs.uk/national-cost-collection/. Accessed 1 Nov 2020

[45] Alva ML, Gray A, Mihaylova B, Leal J, Holman RR (2015). The impact of diabetes-related complications on healthcare costs: new results from the UKPDS (UKPDS 84). Diabet. Med..

[46] Agence technique de l'information sur l'hospitalisation. ENC/T2A: French case-mix-based prospective payment system. https://www.atih.sante.fr/tarifs-mco-et-had. Accessed 23 Nov 2021

[47] Curtis LA, Burns A. Unit Costs of Health and Social Care 2019 Personal Social Services Research Unit (PSSRU). https://www.pssru.ac.uk/project-pages/unit-costs/unit-costs-2019/. Accessed 1 Nov 2020

[48] Decreto Legislativo 1/2005, de 25 de Febrero por el que se Aprueba el Texto Refundido de la Ley de Tasas de la Generalitat. http://www.san.gva.es/documents/151744/2847194/LEY_DE_TASAS_2015.docx. Accessed 1 Sep 2021

[49] Boletín Oficial de Castilla y León. Decreto 25/2010, de 17 de junio, sobre precios públicos por actos asistenciales y servicios sanitarios prestados por la Gerencia Regional de Salud. https://www.saludcastillayleon.es/transparencia/es/transparencia/informacion-datos-publicos/gestion-economica/coste-servicios/precios-publicos-actos-asistenciales-servicios-sanitarios.ficheros/1199560-01%20ANEXO%20PRECIOS%20P%C3%9ABLICOS.pdf. Accessed 21 Sept 2021

[50] Boletín Oficial de la Comunidad de Madrid. Precios publicos por la prestacion de los servicios y actividades de naturaleza sanitaria. http://www.madrid.org/cs/Satellite?blobcol=urldata&blobheader=application%2Fpdf&blobheadername1=Content-Disposition&blobheadervalue1=filename%3DPrecios+p%C3%BAblicos-79942.pdf&blobkey=id&blobtable=MungoBlobs&blobwhere=1352936719948&ssbinary=true. Accessed 1 Sept 2021

[51] Diari Oficial de la Generalitat de Catalunya. ORDEN SLT/71/2020, de 2 de junio, por la que se regulan los supuestos y conceptos facturables y se aprueban los precios públicos correspondientes a los servicios que presta el Instituto Catalán de la Salud. https://portaldogc.gencat.cat/utilsEADOP/PDF/8153/1799007.pdf. Accessed 1 Sept 2021

[52] Osakidetza. Tarifas para Facturacion de Servicios Sanitarios y Docentes de Osakidetza para el Ano 2020. https://www.osakidetza.euskadi.eus/contenidos/informacion/osk_servic_para_empresas/es_def/adjuntos/LIBRO-DE-TARIFAS_2020_osakidetza.pdf. Accessed 1 Sep 2021

[53] Servicio Andaluz de Salud. Precios publicos. https://www.sspa.juntadeandalucia.es/servicioandaluzdesalud/profesionales/recursos-para-profesionales/precios-publicos. Accessed 1 Sep 2021

[54] Chouaid C, Loirat D, Clay E (2017). Cost analysis of adverse events associated with non-small cell lung cancer management in France. Clinicoecon. Outcomes Res..

[55] Torreton E, Vandebrouck T, Emiel P, Detournay B (2013). Cost of inpatient management of hypoglycaemia in France. Value Health..

[56] McMurray JJV, Trueman D, Hancock E (2018). Cost-effectiveness of sacubitril/valsartan in the treatment of heart failure with reduced ejection fraction. Heart.

[57] Escobar C, Varela L, Palacios B (2020). Costs and healthcare utilisation of patients with heart failure in Spain. BMC Health Serv Res..

[58] Sécurité Sociale l'Assurance Maladie. French healthcare insurance: Activité globale et prescriptions des professionnels de santé libéraux. https://assurance-maladie.ameli.fr/etudes-et-donnees/entree-par-theme/professionnels-de-sante-liberaux/activite-globale-et-prescriptions/activite-globale-prescriptions-professionnels-sante-liberaux. Accessed 23 Nov 2021

[59] Sécurité Sociale l'Assurance Maladie. French healthcare insurance: Honoraires des professionnels de santé libéraux. https://assurance-maladie.ameli.fr/etudes-et-donnees/entree-par-theme/professionnels-de-sante-liberaux/honoraires/honoraires-professionnels-sante-liberaux. Accessed 23 Nov 2021

