# Supplementary material for: A European multinational cost-effectiveness analysis of empagliflozin in heart failure with reduced ejection fraction
Source: Eur J Health Econ. 2022 Dec 4;24(9):1441–54. doi: 10.1007/s10198-022-01555-6 (PMC10550866; doi:10.1007/s10198-022-01555-6)
Supplement: Supplementary file 1 — Supplementary file1 (DOCX 231 KB) [file 10198_2022_1555_MOESM1_ESM.docx]

# Supplementary INFORMATION

Title: A European multinational cost-effectiveness analysis of empagliflozin in heart failure with reduced ejection fraction

Author names: Ali Tafazzoli^1^*, Odette S. Reifsnider^1^, Leana Bellanca^2^, Jack Ishak^1^, Marc Carrasco^3^, Pal Rakonczai^4^, Matthew Stargardter^1^, Stephan Linden^5^

Affiliations:

^1^ Evidera, 7101 Wisconsin Avenue, Suite 1400, Bethesda, MD, 20814, USA

^2^ Boehringer Ingelheim Ltd., Ellesfield Avenue, Bracknell, Berkshire, RG12 8YS, UK

^3^ Boehringer Ingelheim España S.A, Prat de la Riba 50, Sant Cugat del Vallès, 08204, Spain

^4^ Evidera, Dorottya Udvar, Bocskai út 134-146-E épület 2. emelet, 1113 Magyarország, Budapest, Hungary

^5^ Boehringer Ingelheim International GmbH, Binger Str. 173, Ingelheim am Rhein, 55216, Germany

*Ali Tafazzoli was an employee of Evidera during the conduct of this study.

Corresponding author e-mail address: [odette.reifsnider@evidera.com](mailto:odette.reifsnider@evidera.com)

## Section S1. Similarities and Differences Between Models for the United Kingdom, Spain, and France

**Table S1. Comparison of United Kingdom, Spain, and France models**

| **Component** | **UK** | **Spain** | **France** |
| --- | --- | --- | --- |
| Population | EMPEROR-Reduced | EMPEROR-Reduced | EMPEROR-Reduced |
| Discount rate, cost and health | 3.5% | 3.0% | 2.5% |
| CV death, all-cause death, HHF | EMPEROR-Reduced patient-level analysis | EMPEROR-Reduced patient-level analysis | EMPEROR-Reduced patient-level analysis |
| Non-CV death | UK national life tables | Spain national life tables | France national life tables |
| Resource use and costs | UK-specific data | Spanish data | French data |
| Treatment discontinuation | Included | Included | Included |
| EQ-5D tariff | van Hout [1] | van Hout [1] | van Hout [1] |
| Cost-effectiveness threshold | £20,000 | €20,000 | €30,000 |

Abbreviations: CV = cardiovascular; HHF = hospitalisation for worsening heart failure; UK = United Kingdom

## Section S2. Statistical Analyses

The economic model tracks the distribution of Kansas City Cardiomyopathy Questionnaire Clinical Summary Score (KCCQ-CSS) health states in the population over time, and updates mortality risk, hospitalisations due to worsening heart failure (HHF), and treatment discontinuation both during and extending beyond the duration of the EMPEROR-Reduced trial. This was accomplished with transition matrices to capture movement between KCCQ-CSS health states and statistical equations that relate treatment allocation and/or current KCCQ-CSS quartile to estimate rate of HHF or time to cardiovascular (CV) death, all-cause death, or treatment discontinuation (Table S2). The effect of KCCQ-CSS state and transient HHF and adverse events (AEs) on health-related quality of life (HRQoL; measured in utilities) was tracked over time in the economic model.

**Transition matrices**

Transition matrices were derived using longitudinal measurements of health states (based on KCCQ-CSS data collected at baseline, week 12, week 32 and week 52, and/or at end of treatment and 30 days thereafter) in the EMPEROR-Reduced trial. KCCQ-CSS was categorised based on quartiles of the observed distribution at baseline with threshold scores of <55, <75, <90, and ≥90. Measurements were organised as one record per visit. Measurements for KCCQ-CSS taken after week 52 were not used in the analyses as the number of available observations beyond this point dropped substantially and exploratory analyses revealed that health states tend to change early on after start of treatment and stabilise fairly early. Imputation was used to handle missing observations over the first 52 weeks (or up to the end of follow-up if the patient died or follow-up ended earlier) using a last-observation carried-forward strategy. Analyses for transition probabilities essentially consisted of deriving the proportion in each health state at the current time stratified by the previous health state; thus, prior levels were retained in the dataset to allow cross-tabulation or use of this as a predictor. Since the derived probabilities represented long-term changes (e.g., 12 weeks, 20 weeks), they were converted to monthly transition probabilities by finding the m-root of the matrices using the *rootm* function in R.

**Rate of HHF**

The occurrence of HHF events was captured using a Poisson model with generalised estimating equations (GEE) with an auto-regressive covariance structure to account for correlations between repeated measures as the data included a record for every month of follow-up for each patient in the trial. Exploratory analyses were carried out, plotting the observed event rates per month in the two study arms to assess the pattern of change over time to determine a suitable parameterisation for time in the equations. In addition to the time parameterisation, the equations included treatment as baseline predictors and time-varying KCCQ-CSS health states were introduced and tested for significance (at p-value of 0.10).

**Time to CV death, all-cause death, or treatment discontinuation**

To obtain the shape of the hazard of CV death and all-cause death, standard parametric survival analysis techniques, as recommended by the Decision Support Unit for the National Institute for Health and Care Excellence [2], were conducted on EMPEROR-Reduced trial patient-level data following two main steps.

- First, a suitable parametric distribution for each event was determined, which included the possibility of selecting different models by treatment arm to account for non-proportionality of effects. Selection of the best fitting parametric model consisted of fitting exponential, Weibull, Gompertz, log-logistic, log-normal, and the generalised gamma distributions to the observed data, and their fit was assessed over the observed data period and beyond to ensure reliable projection. The process involved distinct steps. Diagnostic plots were used to graphically assess fits and provide a preliminary assessment of suitability for each distribution under consideration. Then, numerical fits using Akaike Information Criterion (AIC) and Bayesian Information Criterion (BIC) were compared. Last, the shape of the long-term projection of the curves were assessed to judge the clinical plausibility of extrapolations.
- Second, predictive equations were constructed using the suitable parametric distribution (i.e., building a baseline-only equation, introducing time-varying health states and reducing the model by omitting non-significant predictors [p-value of 0.10]).

Parametric survival analysis was also used to estimate time to treatment discontinuation following the process described above. Treatment discontinuation due to death was not captured in these analyses to avoid double counting with the mortality equations.

**Utilities**

Utility scores were derived from mixed-effects linear regression analysis using all available EQ-5D measurements across all visits of participants in the EMPEROR-Reduced trial.

All patients in the intent-to treat (ITT) population who had a utility measurement available at baseline and at least one other on a later date were considered as eligible for inclusion in the analysis. Time-varying KCCQ-CSS was merged into the dataset to reflect patients’ current health state at the time of each EQ-5D measurement. The statistical model included a random intercept for each patient to account for within-patient correlations. To capture the short- and long-term effects of HHF events on utilities, time varying indicators (1–2, 2–4 and 4–12 months prior to the visit) were created at the time of each EQ-5D measurement to capture time since the hospitalisation. Patients were classified back into the reference group (no HHF) once a year had passed from a hospitalisation – that is, utilities were assumed to return to pre-hospitalisation levels after one year. The effect of AEs was captured the same way but assumed to be more acute. Indicators were created for each type of AE to flag whether it had occurred in the previous month, and patients were returned to the reference group once they were past the first month after the AE. Since the purpose of the analysis included estimating the effect of transient HHF and AE events on utility values, analyses adjusted for other predictors in addition to time-varying KCCQ-CSS that may correlate with these events of interest to isolate the independent effects.

**Table S2. Statistical analyses summary**

| **Outcome** | **Type of statistical analyses** | **Purpose** | **Subgroup-specific** |
| --- | --- | --- | --- |
| Rate of HHF | Poisson model fitted to patient-level data with GEE to account for the repeated measures on patients, adjusting for treatment allocation and time-varying KCCQ-CSS | Used to estimate the incidence of HHF (first and subsequent episodes) | Yes |
| Time to CV death | Parametric survival model adjusting for treatment allocation and time-varying KCCQ-CSS | Used to estimate CV death over time, both during and beyond the duration of EMPEROR-Reduced | Yes |
| Time to all-cause death | Parametric survival model adjusting for treatment allocation and time-varying KCCQ-CSS | Used to estimate all-cause death over time, both during and beyond the duration of EMPEROR-Reduced | Yes |
| Time to treatment discontinuation | Parametric survival model adjusting for treatment allocation and time-varying KCCQ-CSS | Used to estimate discontinuation from treatment with empagliflozin over time, both during and beyond the duration of EMPEROR-Reduced | No |
| Transition probabilities | Longitudinal analysis of KCCQ-CSS data | Used to estimate monthly transition probabilities between health states defined by KCCQ-CSS quartiles | No |
| Utilities | Linear mixed models to account for repeated measures on the same patients, adjusting for KCCQ-CSS, age, sex, region, baseline EQ-5D, medical history, and clinical events | Used to derive health state utility values associated with KCCQ-CSS quartiles and utility decrements associated with incidence of HHF and AEs | No |

Abbreviations: AE = adverse event; CV = cardiovascular; GEE = generalised estimating equation; HHF = hospitalisation for worsening heart failure; KCCQ-CSS = Kansas City Cardiomyopathy Questionnaire Clinical Summary Score

## Section S3. Results of the Statistical Analyses

**Table S3. Estimated risk equation for hospitalisation for worsening heart failure—intention-to-treat population (base case)**

| **Covariate** | **Coefficient (SE)** | **p-value** |
| --- | --- | --- |
| Intercept | -3.347 (0.085) | 0.00000 |
| Empagliflozin treatment | -0.325 (0.097) | 0.00084 |
| KCCQ-CSS: 55 to <75 (Q2)^a^ | -0.450 (0.103) | 0.00001 |
| KCCQ-CSS: 75 to <90 (Q3)^a^ | -0.938 (0.114) | 0.00000 |
| KCCQ-CSS: 90 to 100 (Q4)^a^ | -1.352 (0.142) | 0.00000 |

Poisson model with generalised estimating equations was used to estimate heart failure hospitalisations.

^a^ Relative to KCCQ-CSS: 0 to <55 (Q1)

Abbreviations: KCCQ-CSS = Kansas City Cardiomyopathy Questionnaire Clinical Symptom Score; Q = quartile; SE = standard error

**Table S4. Estimated risk equation for cardiovascular death and all-cause death—intention-to-treat population (base base)**

|  | **CV death** | | **All-cause death** | |
| --- | --- | --- | --- | --- |
| **Covariate** | **Coefficient (SE)** | **p-value** | **Coefficient (SE)** | **p-value** |
| Shape | 1.161 (0.052) | 0.000 | 1.204 (0.047) | 0.000 |
| Scale | 0.000 (0.000)^b^ | 0.003 | 0.000 (0.000)^c^ | 0.001 |
| Empagliflozin treatment | -0.059 (0.101) | 0.562 | -0.044 (0.088) | 0.615 |
| KCCQ-CSS: 55 to <75 (Q2)^a^ | -0.675 (0.129) | 0.000 | -0.609 (0.112) | 0.000 |
| KCCQ-CSS: 75 to <90 (Q3)^a^ | -1.183 (0.143) | 0.000 | -1.161 (0.126) | 0.000 |
| KCCQ-CSS: 90 to 100 (Q4)^a^ | -1.362 (0.148) | 0.000 | -1.299 (0.128) | 0.000 |

Weibull distribution was used to inform the long-term CV death and all-cause death projections.

^a^ Relative to KCCQ-CSS: 0 to <55 (Q1)

^b^ Estimates have been rounded; mean (SE) = 0.00017213 (0.00005835).

^c^ Estimates have been rounded; mean (SE) = 0.00016739 (0.00005102).

Abbreviations: CV = cardiovascular; KCCQ-CSS = Kansas City Cardiomyopathy Questionnaire Clinical Symptom Score; Q = quartile; SE = standard error

**Table S5. Estimated Kansas City Cardiomyopathy Questionnaire Clinical Symptom Score transition probabilities**

| **KCCQ-CSS quartile** | | **Mean** | | | | | |
| --- | --- | --- | --- | --- | --- | --- | --- |
|  |  | **Empagliflozin + SoC** | | | **SoC** | | |
| **From** | **To** | **Month 0–3** | **Month 4–8** | **Month 9+** | **Month 0–3** | **Month 4–8** | **Month 9+** |
| Q1 | Q1 | 0.796 | 0.910 | 0.918 | 0.834 | 0.903 | 0.929 |
|  | Q2 | 0.155 | 0.077 | 0.065 | 0.133 | 0.082 | 0.056 |
|  | Q3 | 0.025 | 0.005 | 0.013 | 0.014 | 0.009 | 0.013 |
|  | Q4 | 0.023 | 0.008 | 0.004 | 0.018 | 0.005 | 0.002 |
| Q2 | Q1 | 0.066 | 0.068 | 0.051 | 0.069 | 0.058 | 0.051 |
|  | Q2 | 0.708 | 0.840 | 0.881 | 0.720 | 0.849 | 0.867 |
|  | Q3 | 0.188 | 0.083 | 0.061 | 0.203 | 0.079 | 0.076 |
|  | Q4 | 0.038 | 0.009 | 0.007 | 0.008 | 0.013 | 0.006 |
| Q3 | Q1 | 0.004 | 0.005 | 0.004 | 0.013 | 0.011 | 0.013 |
|  | Q2 | 0.082 | 0.070 | 0.054 | 0.112 | 0.058 | 0.054 |
|  | Q3 | 0.771 | 0.848 | 0.868 | 0.743 | 0.859 | 0.871 |
|  | Q4 | 0.142 | 0.077 | 0.074 | 0.132 | 0.072 | 0.062 |
| Q4 | Q1 | 0.006 | 0.004 | 0.003 | 0.006 | 0.004 | 0.000 |
|  | Q2 | 0.016 | 0.000 | 0.006 | 0.009 | 0.008 | 0.005 |
|  | Q3 | 0.074 | 0.063 | 0.044 | 0.096 | 0.058 | 0.049 |
|  | Q4 | 0.904 | 0.933 | 0.947 | 0.889 | 0.931 | 0.945 |

Abbreviations: KCCQ-CSS = Kansas City Cardiomyopathy Questionnaire Clinical Symptom Score; Q = quartile; SoC = standard of care

**Table S6. Estimated risk equation for treatment discontinuation**

| **Covariate** | **Coefficient (SE)** | **p-value** |
| --- | --- | --- |
| Rate | 0.001 (0.000)^b^ | 0.000 |
| KCCQ-CSS: 55 to <75 (Q2)^a^ | -0.344 (0.128) | 0.007 |
| KCCQ-CSS: 75 to <90 (Q3)^a^ | -0.881 (0.139) | 0.000 |
| KCCQ-CSS: 90 to 100 (Q4)^a^ | -1.083 (0.141) | 0.000 |

Exponential distribution was used to inform treatment discontinuation.

^a^ Relative to KCCQ-CSS: 0 to <55 (Q1)

^b^ Estimate have been rounded; SE = 0.00008082.

Abbreviations: KCCQ-CSS = Kansas City Cardiomyopathy Questionnaire Clinical Symptom Score; Q = quartile; SE = standard error

**Table S7. Estimated utility equations from linear mixed models**

| **Coefficient** | **UK** | | **Spain** | | **France** | |
| --- | --- | --- | --- | --- | --- | --- |
|  | **Coefficient (SE)** | **p-value** | **Coefficient (SE)** | **p-value** | **Coefficient (SE)** | **p-value** |
| Intercept | 0.591 (0.007) | <0.001 | 0.603 (0.007) | 0.000 | 0.488 (0.008) | 0.000 |
| Sex: male | 0.019 (0.005) | <0.001 | 0.015 (0.005) | 0.001 | 0.016 (0.006) | 0.004 |
| Age ≥65 years | -0.008 (0.004) | 0.038 | -0.010 (0.004) | 0.019 | -0.014 (0.005) | 0.003 |
| Region: Asia | 0.000 (0.006) | 0.995 | -0.003 (0.006) | 0.688 | -0.003 (0.007) | 0.724 |
| Region: Latin America | 0.017 (0.005) | <0.001 | 0.019 (0.005) | 0.000 | 0.027 (0.006) | 0.000 |
| Region: North America | 0.022 (0.007) | <0.001 | 0.023 (0.007) | 0.001 | 0.029 (0.008) | 0.000 |
| Region: other | 0.038 (0.010) | <0.001 | 0.043 (0.010) | 0.000 | 0.053 (0.012) | 0.000 |
| Baseline EQ-5D (standardised) | 0.082 (0.002) | <0.001 | 0.088 (0.002) | 0.000 | 0.102 (0.003) | 0.000 |
| Ischaemic HF | -0.013 (0.004) | 0.002 | -0.013 (0.004) | 0.001 | -0.015 (0.005) | 0.002 |
| HF hospitalisation: <1 month | -0.032 (0.012) | 0.007 | -0.046 (0.012) | 0.000 | -0.053 (0.014) | 0.000 |
| HF hospitalisation: 1 to <2 months | -0.040 (0.011) | <0.001 | -0.045 (0.011) | 0.000 | -0.045 (0.013) | 0.000 |
| HF hospitalisation: 2 to <4 months | -0.020 (0.010) | 0.003 | -0.026 (0.010) | 0.007 | -0.026 (0.011) | 0.019 |
| HF hospitalisation: 4 to <12 months | -0.015 (0.007) | 0.040 | -0.018 (0.007) | 0.010 | -0.011 (0.008) | 0.178 |
| KCCQ-CSS: 55 to <75 (Q2) | 0.117 (0.005) | <0.001 | 0.125 (0.005) | 0.000 | 0.146 (0.005) | 0.000 |
| KCCQ-CSS: 75 to <90 (Q3) | 0.190 (0.005) | <0.001 | 0.203 (0.005) | 0.000 | 0.256 (0.006) | 0.000 |
| KCCQ-CSS: 90 to 100 (Q4) | 0.254 (0.005) | <0.001 | 0.262 (0.005) | 0.000 | 0.346 (0.006) | 0.000 |
| Urinary tract infection | 0.000 (0.019) | 0.998 | 0.002 (0.019) | 0.920 | 0.006 (0.023) | 0.797 |
| Genital mycotic infection | -0.058 (0.039) | 0.134 | -0.053 (0.039) | 0.171 | -0.052 (0.045) | 0.254 |
| Acute renal failure | -0.010 (0.012) | 0.399 | -0.014 (0.012) | 0.247 | -0.011 (0.014) | 0.445 |
| Hepatic injury | -0.016 (0.019) | 0.398 | -0.011 (0.019) | 0.573 | -0.018 (0.022) | 0.410 |
| Volume depletion | -0.018 (0.014) | 0.206 | -0.015 (0.014) | 0.283 | -0.017 (0.017) | 0.316 |
| Hypoglycaemic event | -0.048 (0.033) | 0.150 | -0.041 (0.034) | 0.218 | -0.055 (0.039) | 0.154 |
| Bone fracture | -0.165 (0.036) | <0.001 | -0.170 (0.036) | 0.000 | -0.148 (0.041) | 0.000 |

Linear mixed models were used to estimate utility equations.

Abbreviations: HF = heart failure; KCCQ-CSS = Kansas City Cardiomyopathy Questionnaire clinical symptom score; Q = quartile; SE = standard error; UK = United Kingdom

## Section S4. Adverse Events

**Table S8. Adverse event rates**

|  | **Rate per 100 PYs** | |  |
| --- | --- | --- | --- |
|  | **UK, Spain and France** | |  |
| **AE** | **Empagliflozin + SoC** | **SoC** | **Source** |
| Urinary tract infection | 4.13 | 3.76 | EMPEROR-Reduced |
| Genital mycotic infection | 1.38 | 0.53 |  |
| Acute renal failure | 8.13 | 9.02 |  |
| Hepatic injury | 3.43 | 3.83 |  |
| Volume depletion | 9.26 | 8.76 |  |
| Hypotension | 8.22 | 7.69 |  |
| Hypoglycaemic event | 1.20 | 1.25 |  |
| Bone fracture | 2.01 | 1.89 |  |

Abbreviations: AE = adverse event; PY = patient-year; SoC = standard of care; UK = United Kingdom

## Section S5. Utility Inputs

**Table S9. Utility inputs estimated from linear mixed effect regression models**

|  | **Mean (SE)** | | |  |  |
| --- | --- | --- | --- | --- | --- |
| **Parameter** | **UK** | **Spain** | **France** | **Duration** | **Source** |
| **Health state utility** | | | | | |
| KCCQ-CSS: 0 to <55 (Q1)^a^ | 0.520 (0.004) | 0.629 (0.004) | 0.464 (0.005) | Permanent | EMPEROR Reduced |
| KCCQ-CSS: 55 to <75 (Q2)^a^ | 0.637 (0.003) | 0.754 (0.003) | 0.610 (0.004) | Permanent | EMPEROR Reduced |
| KCCQ-CSS: 75 to <90 (Q3)^a^ | 0.710 (0.003) | 0.832 (0.003) | 0.721 (0.004) | Permanent | EMPEROR Reduced |
| KCCQ-CSS: 90 to 100 (Q4)^b^ | 0.774 (0.003) | 0.891 (0.003) | 0.810 (0.004) | Permanent | Sullivan et al. 2011 (UK) [3]; Szende et al. 2014 (Spain) [4]; Janssen and Szende 2014 (France) [5] |
| **Clinical event disutility** | | | | | |
| HHF | -0.246 (0.062) | -0.291 (0.062) | -0.240 (0.072) | 1 month | EMPEROR Reduced |
| Urinary tract infection | -0.025 (0.027) | -0.025 (0.027) | -0.025 (0.027) | 1 month | Sullivan and Ghushchyan 2016 [6] |
| Genital mycotic infection | -0.058 (0.039) | -0.053 (0.039) | -0.052 (0.045) | 1 month | EMPEROR Reduced |
| Acute renal failure | -0.010 (0.012) | -0.014 (0.012) | -0.011 (0.014) | 1 month | EMPEROR Reduced |
| Hepatic injury | -0.016 (0.019) | -0.011 (0.019) | -0.018 (0.019) | 1 month | EMPEROR Reduced |
| Volume depletion | -0.018 (0.014) | -0.016 (0.014) | -0.017 (0.014) | 1 month | EMPEROR Reduced |
| Hypotension | -0.025 (0.000) | -0.025 (0.000) | -0.025 (0.000) | 1 month | Sullivan and Ghushchyan 2006 [7]^c^ |
| Hypoglycaemic event | -0.048 (0.033) | -0.041 (0.034) | -0.055 (0.039) | 1 month | EMPEROR Reduced |
| Bone fracture | -0.165 (0.035) | -0.170 (0.036) | -0.148 (0.036) | 1 month | EMPEROR Reduced |

Utility values were estimated from the linear mixed effect regression models for the UK, Spain, and France.

^a^ Relative differences from the EMPEROR-Reduced study were applied to general population utility for people in the UK aged 60–69 [3], Spain aged 65–74 [4], and France aged 65–74 [5].

^b^ Assumed equal to the general population utility value for people in the UK aged 60–69 [3], Spain aged 65–74 [4], and France aged 65–74 [5]

^c^ Assumed same as essential hypertension

Abbreviations: HHF = hospitalisation for worsening heart failure; KCCQ-CSS = Kansas City Cardiomyopathy Questionnaire clinical symptom score; Q = quartile; SE = standard error; UK = United Kingdom

## Section S6. Cost Inputs

### **UK setting**

**Table S10. Estimated drug cost by treatment regimen in the United Kingdom (2021 British pounds)**

|  | **Monthly Cost (utilisation in EMPEROR-Reduced)** | | **Source** |
| --- | --- | --- | --- |
| **Treatment regimen** | **Empagliflozin + SoC** | **SoC** | MIMS Drug Database [8] |
| Empagliflozin | £39.78 (100%) | – |  |
| ACEi | £6.03 (45%) | £6.03 (45%) |  |
| ARB | £11.05 (24%) | £11.05 (24%) |  |
| ARNi | £99.53 (20%) | £99.53 (20%) |  |
| MRA | £7.63 (71%) | £7.63 (71%) |  |
| BB | £9.50 (95%) | £9.50 (95%) |  |
| Ivabradine | £4.83 (7%) | £4.83 (7%) |  |
| Estimated weighted drug cost per month^a^ | £79.38 | £39.60 |  |

Costs were calculated for each treatment class using monthly treatment costs estimated from the dosage of each drug and number of doses per month for each treatment based on the summary of product characteristics and assuming individual therapies within each class are uniformly distributed.

^a^ Cost applied in the base case

Abbreviations: ACEi = angiotensin-converting enzyme inhibitor; ARB = angiotensin receptor blocker; ARNi = angiotensin receptor neprilysin inhibitor; BB = beta blocker; MIMS = Monthly Index of Medical Specialties; MRA = mineralocorticoid receptor antagonist; SoC = standard of care

**Table S11. Acute cost related to clinical events in the United Kingdom (2021 British pounds)**

| **Event** | **Cost per event** | **HRG code** | **Source** |
| --- | --- | --- | --- |
| HHF | £3,072 | EB03A-E, heart failure or shock, non-elective long stay | NHS 2018–2019 [9] |
| CV death | £4,146^a^ | NA | Alva et al. 2015 [10] |
| Non-CV death | £0 | NA | Assumption |
| Urinary tract infection | £40 | 10.3b^b^ | PSSRU 2020 [11] |
| Genital mycotic infection | £40 | 10.3b^b^ | PSSRU 2020 [11] |
| Acute renal failure | £1,906 | LA07H, LA07J-N, LA07P, acute kidney injury, non-elective long or short stay^c^ | NHS 2018–2019 [9] |
| Hepatic injury | £1,274, computed from £40 (50%) and £2,508 (50%) | 10.3b^b^; GC01C-F, liver failure disorders, non-elective long or short stay^c^ | PSSRU 2020 [11]; NHS 2018–2019 [9] |
| Volume depletion | £40 | 10.3b^b^ | PSSRU 2020 [11] |
| Hypotension | £40 | 10.3b^b^ | PSSRU 2020 [11] |
| Hypoglycaemic event | £627, computed from £40 (50%) and £1,213 (50%) | 10.3b*; KA08A-C, other endocrine disorders, non-elective long or short stay^c^ | PSSRU 2020 [11]; NHS 2018–2019 [9] |
| Bone fracture | £2,710 | HD39D-H, pathological fractures, non-elective long or short stay^c^ | NHS 2018–2019 [9] |

^a^ Reflects the average of the cost of fatal myocardial infarction, fatal ischaemic heart disease, and fatal stroke, applying the coefficients of the regression equation reported in Table 2 in Alva et al. (2015) [10].

^b^ Per patient contact lasting 9.22 minutes.

^c^ Weighted mean by national average unit costs and number of finished consultant episodes

Abbreviations: CV = cardiovascular; HHF = hospitalisation for worsening heart failure; HRG = Healthcare Resource Group; NA = not applicable; NHS = National Health Service; PSSRU = Personal Social Services Research Unit

**Table S12. Disease management cost for Kansas City Cardiomyopathy Questionnaire Clinical Summary Score in the United Kingdom (2021 British pounds)**

|  | **KCCQ-CSS quartile 1–4** | | | | |
| --- | --- | --- | --- | --- | --- |
|  | **Monthly frequency** | **Source** | **Monthly cost** | **Medical code / description** | **Source** |
| GP visit | 1.9283 | McMurray et al. 2018 [12] | £40 | 10.3b^a^ | PSSRU 2020 [11] |
| Cardiologist visit | 0.0042 | McMurray et al. 2018 [12] | £140 | Cardiology - non-admitted, face to face, follow-up visit | NHS 2018-2019 [9] |
| A&E referral | 0.0008 | McMurray et al. 2018 [12] | £154 | VB01Z-VB11Z, VB99Z^b^ | NHS 2018-2019 [9] |
| Estimated total monthly cost per patient | £77 | | | | |

Abbreviations: A&E = accident and emergency; GP = general practitioner; KCCQ-CSS = Kansas City Cardiomyopathy Questionnaire Clinical Summary Score; NHS = National Health Service; PSSRU = Personal Social Services Research Unit

^a^ Per patient contact lasting 9.22 minutes.

^b^ Weighted mean by national average unit costs and number of finished consultant episodes for emergency medicine.

### **Spanish setting**

**Table S13. Estimated drug cost by treatment regimen in Spain (2021 Euros)**

|  | **Monthly Cost (utilisation in EMPEROR-Reduced)** | | **Source** |
| --- | --- | --- | --- |
| **Treatment regimen** | **Empagliflozin + SoC** | **SoC** | BotPlus Web Database [13] |
| Empagliflozin | €56.26 (100%) | – |  |
| ACEi | €7.30 (45%) | €7.30 (45%) |  |
| ARB | €23.94 (24%) | €23.94 (24%) |  |
| ARNi | €210.18 (20%) | €210.18 (20%) |  |
| MRA | €54.12 (71%) | €54.12 (71%) |  |
| BB | €8.87 (95%) | €8.87 (95%) |  |
| Ivabradine | €47.04 (7%) | €47.04 (7%) |  |
| Estimated weighted drug cost per month^a^ | €156.66 | €100.40 |  |

Costs were calculated for each treatment class using monthly treatment costs estimated from the dosage of each drug and number of doses per month for each treatment and assuming individual therapies within each class are uniformly distributed.

^a^ Cost applied in the base case.

Abbreviations: ACEi = angiotensin-converting enzyme inhibitor; ARB = angiotensin receptor blocker; ARNi = angiotensin receptor neprilysin inhibitor; BB = beta blocker; MRA = mineralocorticoid receptor antagonist; SoC = standard of care

**Table S14. Acute cost related to clinical events in Spain (2021 Euros)**

| **Event** | **Cost per event** | **ICD-10 code** | **Source** |
| --- | --- | --- | --- |
| HHF | €3,814 | 150 | Ministerio de Sanidad, Consumo y Bienestar Social 2021 [14] |
| CV death | €6,276^a^ | 121, 163 |  |
| Non-CV death | €0 | NA | Assumption |
| Urinary tract infection | €57 | NA | Varied sources [15-20]^b^ |
| Genital mycotic infection | €57 | NA | Varied sources [15-20]^b^ |
| Acute renal failure | €4,243 | N17 | Ministerio de Sanidad, Consumo y Bienestar Social 2018 [14] |
| Hepatic injury | €2,709, estimated from €57 (50%) and €5,362 (50%) | NA; K72 | Varied sources [15-20]^b^; Ministerio de Sanidad, Consumo y Bienestar Social 2018 [14] |
| Volume depletion | €57 | NA | Varied sources [15-20]^b^ |
| Hypotension | €57 | NA | Varied sources [15-20]^b^ |
| Hypoglycaemic event | €1,461, estimated from €57 (50%) and €2,865 (50%) | NA; E16 | Varied sources [15-20]^b^; Ministerio de Sanidad, Consumo y Bienestar Social 2018 [14] |
| Bone fracture | €5,042 | S82 | Ministerio de Sanidad, Consumo y Bienestar Social 2018 [14] |

^a^ Reflects data extracted from the Ministerio de Sanidad Consumo y Bienestar Social discharge registry, involving fatal events associated with ICD-10 codes I21 (acute myocardial infarction) and I63 (cerebral infarction) [14].

^b^ Average first and subsequent general practitioner visits, average for 6 regions in Spain (Madrid, Catalonia, Andalusia, Basque Country, Valencia, Castilla León)

Abbreviations: CV = cardiovascular; HHF = hospitalisation for worsening heart failure; ICD-10 = International Classification of Diseases, 10th Revision; NA = not applicable

**Table S15. Disease management cost for Kansas City Cardiomyopathy Questionnaire Clinical Summary Score in Spain (2021 Euros)**

|  | **KCCQ-CSS quartile 1–4** | | | |
| --- | --- | --- | --- | --- |
|  | **Monthly frequency** | **Source** | **Monthly cost** | **Source/description** |
| GP visit | 0.6417 | Escobar et al. 2020 [21] | €57 | Varied sources [15-20]^a^ |
| Cardiologist visit | 0.0917 |  | €115 |  |
| A&E referral | 0.0417 |  | €196 |  |
| Estimated total monthly cost per patient | €55 | | | |

^a^ Average first and subsequent general practitioner visits, average for 6 regions in Spain (Madrid, Catalonia, Andalusia, Basque Country, Valencia, Castilla León)

Abbreviations: A&E = accident and emergency; GP = general practitioner; KCCQ-CSS = Kansas City Cardiomyopathy Questionnaire Clinical Summary Score

### **French setting**

**Table S16. Estimated drug cost by treatment regimen in France (2021 Euros)**

|  | **Monthly Cost (utilisation in EMPEROR-Reduced)** | | **Source** |
| --- | --- | --- | --- |
| **Treatment regimen** | **Empagliflozin + SoC** | **SoC** | Official Journal [22] |
| Empagliflozin | €45.63 (100%) | – |  |
| ACEi | €12.11 (45%) | €12.11 (45%) |  |
| ARB | €15.14 (24%) | €15.14 (24%) |  |
| ARNi | €160.74 (20%) | €160.74 (20%) |  |
| MRA | €37.15 (71%) | €37.15 (71%) |  |
| BB | €16.00 (95%) | €16.00 (95%) |  |
| Ivabradine | €23.15 (7%) | €23.15 (7%) |  |
| Estimated weighted drug cost per month^a^ | €129.41 | €83.79 |  |

Costs were calculated for each treatment class using monthly treatment costs estimated from the dosage of each drug and number of doses per month for each treatment based on ESC 2016 guidelines (Metoprolol) [23] or SmPC (other drugs) [24] and applying a distribution for individual therapies within each class. SGLT2i: empagliflozin (100%); ACEi: captopril (1%), enalapril (9%), lisinopril (4%), ramipril (84%), trandolapril (2%); ARB: candesartan (68.16%), valsartan (15.40%), losartan (16.44%); ARNi: sacubitril / valsartan (100%); MRA: eplerenone (100%); BB: bisoprolol (70%), carvedilol (1%), metoprolol (0%), nebivolol (29%); and HCN channel blocker: ivabradine (100%). Costs are official journal prices (public prices including all taxes) and include a packaging fee (€1.02), dispensation fee (€0.51), age-related fee (€1.58), and dispensing fee (€3.57).

^a^ Cost applied in the base case.

Abbreviations: ACEi = angiotensin-converting enzyme inhibitor; ARB = angiotensin receptor blocker; ARNi = angiotensin receptor neprilysin inhibitor; BB = beta blocker; MRA = mineralocorticoid receptor antagonist; SoC = standard of care

**Table S17. Acute cost related to clinical events in France (2021 Euros)**

| **Event** | **Cost per event** | **Medical code** | **Source** |
| --- | --- | --- | --- |
| HHF | €4,968 | ICD-10 code: I50 | Entresto efficiency report [25] |
| CV death | €3,764^a^ | GHM: 23Z02T/Z | ENC/T2A: French case-mix-based prospective payment system [26] |
| Non-CV death | €0 | NA | Assumption |
| Urinary tract infection | €36 | NA | Entresto's efficiency report [25] |
| Genital mycotic infection | €36 | NA | Entresto's efficiency report [25] |
| Acute renal failure | €3,739 | NA | Chouaid et al. 2017 [27] |
| Hepatic injury | €1,055, estimated from €36 (50%) and €2,074 (50%) | NA; K711 | Entresto's efficiency report [25]; ENC / T2A : French case-mix-based prospective payment system [26] |
| Volume depletion | €36 | NA | Entresto's efficiency report [25] |
| Hypotension | €36 | NA | Entresto's efficiency report [25] |
| Hypoglycaemic event | €2,319, estimated €36 (50%) and €4,602 (50%) | NA; NA | Entresto's efficiency report [25]; Torreton et al. 2013 [28] |
| Bone fracture | €3,352 | S729 | ENC / T2A : French case-mix-based prospective payment system [26] |

^a^ Reflects national cost scale activity pricing established for the French case-mix-based prospective payment system for the delivery of palliative care (GHM: 23Z02T/Z) [26].

Abbreviations: CV = cardiovascular; GHM = Groupes homogènes de maladies; HHF = hospitalisation for worsening heart failure; ICD-10 = International Classification of Diseases, 10th Revision; NA = not applicable

**Table S18. Disease management cost for Kansas City Cardiomyopathy Questionnaire Clinical Summary Score in France (2021 Euros)**

|  | **KCCQ-CSS quartile 1–4** | | | |
| --- | --- | --- | --- | --- |
|  | **Monthly frequency** | **Source/description** | **Monthly cost** | **Source/description** |
| GP visit | 0.7758 | Entresto efficiency report [25] | €36 | French healthcare insurance Ameli et al. 2018 [29, 30] |
| Cardiologist visit | 0.0558 |  | €243 |  |
| A&E referral | 0.0417 | Escobar et al. 2020 [21]; assumed similar to Spain | €151 | French general accounting office 2019 report on hospital emergencies [6] |
| Estimated total monthly cost per patient | €48 | | | |

Abbreviations: A&E = accident and emergency; GP = general practitioner; KCCQ-CSS = Kansas City Cardiomyopathy Questionnaire Clinical Summary Score

**Table S19. Outline of treatment cost calculations**

|  | **UK** | **Spain** | **France** |
| --- | --- | --- | --- |
| (a) Package cost of empagliflozin | £36.59 | €55.45 | €38.29 |
| (b) Package size | 28 pills | 30 pills | 30 pills |
| (c) Daily cost (administered once daily) [a / b] | £1.31 | €1.85 | €1.50 (includes €0.22 in packaging and dispensing fees) |
| (d) Annual cost of empagliflozin [d * 365.25] | £477.30 | €675.10 | €547.51 |
| (e) Annual cost of SoC | £475.23 | €1,204.77 | €1,005.47 |
| (f) Average time on treatment | Undiscounted: 3.45 years  Discounted: 3.10 years | Undiscounted 3.46 years  Discounted: 3.15 years | Undiscounted: 3.46 years  Discounted: 3.19 years |
| (g) Average life expectancy | Undiscounted: 6.99 years  Discounted: 5.81 years | Undiscounted: 7.01 years  Discounted: 5.96 years | Undiscounted: 7.01 years  Discounted: 6.11 years |
| **Average lifetime costs (undiscounted)** | | | |
| (h) Average lifetime cost of empagliflozin [d * f] | £1,649.06 | €2,332.98 | €1,891.98 |
| (i) Average lifetime cost of SoC [e * g] | £3,323.63 | €8,445.05 | €7,049.67 |
| (j) Average total treatment cost [h + i] | £4,972.69 | €10,778.03 | €8,941.65 |
| **Average lifetime costs (discounted)^a^** | | | |
| (k) Average lifetime cost of empagliflozin [d * f] | £1,480.72 | €2,125.32 | €1,749.26 |
| (l) Average lifetime cost of SoC [e * g] | £2,759.28 | €7,178.60 | €6,145.44 |
| (m) Total [k + l] | £4,240.00 | €9,303.92 | €7,894.69 |

^a^ These values are calculated by multiplying treatment costs in each year by a discount factor, and then summing the resultant figures over the model horizon.

Abbreviations: SoC = standard of care; UK = United Kingdom

## Section S7. Validation

**Table S20. Comparison of observed and model-predicted event rates, intention-to-treat population**

|  | **Model predicted event rate 100 PY** | | **EMPEROR-Reduced event rate per 100 PY (95% CI)** | |
| --- | --- | --- | --- | --- |
| **Event** | **Empa + SoC** | **SoC** | **Empa + SoC** | **SoC** |
| HHF | 15.97 | 20.82 | 15.77 (14.19, 17.31) | 22.44 (20.49, 24.20) |
| CV death | 7.10 | 7.80 | 7.55 (6.54, 8.72) | 8.13 (7.08, 9.33) |
| All-cause death | 9.41 | 10.19 | 10.06 (8.88, 11.38) | 10.71 (9.49, 12.71) |

Abbreviations: CI = confidence interval; CV = cardiovascular; Empa = empagliflozin; HHF = hospitalisation for worsening heart failure; PY = patient-year; SoC = standard of care

**Generalisability of EMPEROR-Reduced baseline characteristics to real-world evidence (RWE)**

To assess the generalisability of EMPEROR-Reduced trial to clinical practice in the UK, Spain, and France, participant baseline characteristics and clinical outcomes were compared with those from patients involved in the Incidence Prevalence and resoUrce utiLiSation of hEart failure in England (PULSE) study, PATHWAYS-HF in Spain , and the FREnch Survey on Heart Failure (FRESH):

- PULSE was a non-interventional cohort study that included English adults diagnosed with chronic heart failure (HF) in primary or secondary care between calendar years 2015 through 2019, inclusive, and who contributed to the UK Clinical Practice Research Datalink Aurum dataset, which was then linked to inpatient and mortality data extracted from Hospital Episodes Statistics and the Office for National Statistics, respectively. This comparison focuses on the subset of PULSE participants with reduced ejection fraction (*N* = 68,780). As reported in Table S21, PULSE participants were comparatively older, less diverse, and more likely to be female than patients taking part in EMPEROR-Reduced, had been diagnosed more recently, and were less likely to possess baseline type 2 diabetes mellitus (T2DM) or to have been hospitalised for HF in the previous year. They also tended to have higher body mass index (BMI), blood pressure, heart rate, left ventricular ejection fraction, baseline kidney function, and likelihood of prior atrial fibrillation or flutter and HF from ischemic cause, but lower mean and median N-terminal pro B-type natriuretic peptide (NT-proBNP). Finally, they were about equally likely to have implantable cardioverter-defibrillators or to use ACE or ARBs, but were less commonly administered MRAs, BBs, or ARNI. Due to significant gaps in PULSE data (95% or more missing for some key baseline characteristics), it is difficult to establish the representativeness of PULSE participants—and, by extension, the generalisability of the attributes of patients enrolled in EMPEROR-Reduced—to clinical practice in the UK.
- PATHWAYS-HF is a retrospective observational study designed to better understand the epidemiology, characteristics, and treatment of adult patients with HF in Spain through analysis of electronic medical records extracted from the BIG-PAC administrative database; of note, a previously-undertaken demographic comparison verified the representativeness of the BIG-PAC data to the Spanish population more broadly [31]. This comparison focuses on the subset of study participants with chronic HF characterised by reduced ejection fraction (i.e., LVEF ≤ 40%) (*N* = 10,217) during the period between 2017 and 2019, inclusive. As indicated in Table S21, PATHWAYS-HF participants tended to be older than patients taking part in EMPEROR-Reduced and included a somewhat larger proportion of females. They were also less likely to be concurrently managing T2DM, to possess HF of ischemic etiology, or to have a record of prior atrial fibrillation or flutter, but the severity of their disease was greater, on average (i.e., a larger proportion with New York Heart Association [NYHA] Class III/IV). Information regarding other characteristics of interest (e.g., baseline BMI, mean and median NT-proBNP, prior hospitalisation for HF, etc.) was unavailable. Patients in PATHWAYS-HF were also less likely to utilise ACE, ARBs, MRAs, BBs, or ivabradine, but were administered ARNI at roughly comparable rates.
- FRESH is a longitudinal, multicentre study administered by the Société Française de Cardiologie that consists of patients aged 18 or older diagnosed with chronic HF or previously hospitalised with acute HF [32]. This comparison focuses on the subset of FRESH participants with chronic HF characterised by reduced ejection fraction (*N* = 904). As shown in Table S21, this subset of patients generally resembled EMPEROR-Reduced participants, although the former was slightly younger, comparatively less likely to have T2DM or arterial hypertension at baseline, and had more severe disease, on average (i.e., a larger proportion with NYHA Class III/IV). Information regarding some patient characteristics was unavailable, as was data regarding baseline treatment utilisation.

**Table S21. Comparison of baseline patient characteristics in EMPEROR-Reduced vs. real-world studies**

|  | **EMPEROR-Reduced (N = 3,730)** | **UK (PULSE) (N = 68,780)** | **Spain (PATH-WAYS-HF)**  **(N = 10,217)** | **France (FRESH) (N = 904)** |
| --- | --- | --- | --- | --- |
| **Demographics** | | | | |
| Age | 66.8 | 72.2 | 73.2 | 63.5 |
| Race: White | 70.5% | 91.8% | NA | NA |
| Sex: Male | 76.1% | 65.1% | 69.0% | 77% |
| **Medical History** | | | | |
| Time since diagnosis (years) | 6.14 | 3.8 | NA | NA |
| Has type 2 diabetes mellitus (T2DM) | 49.8% | 25.9% | 31.1% | 28.7% |
| HF: Ischemic cause | 51.7% | 67.8% | 42.5% | NA |
| Body mass index (BMI; kg/m^2^) | 27.9 | 28.5 | NA | 27 |
| Heart rate (HR; beats/min) | 71.3 | 74.6 | NA | 72 |
| Left ventricular ejection fraction (LVEF) (%) | 26.8 | 32.1 | NA | 29 |
| eGFR at baseline >= 60 ml/min/1.73m2 (%) | 51.8% | 64.5% | NA | NA |
| Prior hospitalisation for HF in the previous 12 months (%) | 30.9% | 5.9% | NA | NA |
| Prior atrial fibrillation or flutter (%) | 38.6% | 44.2% | 23.4% | 38.1% |
| NT-proBNP (pg/mL; mean) | 3,034.7 | 1989.3 | NA | NA |
| NT-proBNP (pg/mL; median) | 1,910 | 1,063 | NA | NA |
| Arterial hypertension at baseline (%) | 72.3% | NA | 65.4% | 47.4% |
| Systolic blood pressure (SBP; mmHg) | 122.0 | 127.8 | NA | 118 |
| NYHA III or IV (%) | 24.9% | NA | 49.5% | 34% |
| **Treatment use at baseline** | | | | |
| Implantable cardioverter-defibrillator (%) | 22.8% | 21.7% | NA | NA |
| ACE or ARBs (%) | 69.7% | 73.3% | 64.5%^a^ | NA |
| MRA (%) | 71.3% | 20.8% | 29.8%^a^ | NA |
| BB (%) | 94.7% | 65.3% | 64.3%^a^ | NA |
| ARNI (%) | 19.5% | 0.3% | 16.0%^a^ | NA |
| Ivabradine (%) | 7.0% | NA | 2.3%^a^ | NA |

^a^ Referred to as “atrial fibrillation” in the original source (i.e., no explicit reference to flutter); ^b^ Reflects utilisation rates reported for 2019 in Sicras-Mainar et al. (2022) [31]; ^c^ Referred to as aldosterone antagonists in the original source.

Abbreviations: ACE = angiotensin-converting enzyme; ARB = Angiotensin II receptor blocker; ARNI = angiotensin Receptor-Neprilysin Inhibitor; BB = beta blocker; BMI = body mass index; eGFR = estimated glomerular filtration rate; FRESH = FREnch Survey on Heart Failure; HF = heart failure; LVEF = left ventricular ejection fraction; MRA = mineralocorticoid receptor antagonist; NA = not available; NT-proBNP = N-terminal pro-brain natriuretic peptide; NYHA = New York Heart Association; PULSE = Incidence Prevalence and resoUrce utiLiSation of hEart failure in England; SBP = systolic blood pressure; T2DM = type 2 diabetes mellitus; UK = United Kingdom

**Generalisability of EMPEROR-Reduced trial outcomes to real-world evidence (RWE)**

- In PULSE, age- and sex-adjusted HHF rates were significantly lower than the placebo arm of EMPEROR-Reduced (10.2 vs. 22.3/100 person-years [PY], respectively), while rates of CV and all-cause mortality were slightly lower and higher, respectively (CV: 6.1 vs. 8.1/100 PY; all-cause: 12.7 vs. 10.7/100 PY). Noting the comparability of mortality rates, however, it is likely that observed differences in HHF rates reflect underlying differences in how these events were recorded.
- Although Sicras-Mainar et al. [31] provide limited reporting on clinical outcomes in HFrEF patients participating in PATHWAYS-HF, a lack of detail regarding the methods used in their derivation (most notably, the time interval to which they apply) precludes comparison with EMPEROR-Reduced. A sub-analysis conducted in a related study involving the same data source and an HFrEF population satisfying DAPA-HF inclusion criteria (*N* = 4,243) estimated a one-year all-cause mortality rate of 12.2% [33], which is reasonably comparable to the rate of 10.7% observed in EMPEROR-Reduced (see Table S22 below); however, given the criteria used to construct the sample, the generalisability of this result to the broader population of Spanish HFrEF patients is uncertain.
- As illustrated in Table S22, in FRESH rates of CV hospitalisation and all-cause mortality at two years were somewhat lower than the rates observed in EMPEROR-Reduced, whereas one-year rates of all-cause mortality were similar:

**Table S22. Comparison of clinical event rates, EMPEROR-Reduced vs. FRESH**

| **Clinical Event** | **EMPEROR-Reduced (Placebo; N = 1,867)** | **France (FRESH)**  **(N = 904)** |
| --- | --- | --- |
| CV hospitalisation rate at 2 years (%) | 23% | 18% |
| All-cause mortality rate at 1 year (%) | 10.7% | 9.9% |
| All-cause mortality rate at 2 years (%) | 20.0% | 15.7% |

Abbreviations: CV = cardiovascular; FRESH = FREnch Survey on Heart Failure

## Section S8. Supplementary Base-Case Results

**Table S23. Base-case results, disaggregated by KCCQ-CSS quartile**

|  | **UK** | | | **Spain** | | | **France** | | |
| --- | --- | --- | --- | --- | --- | --- | --- | --- | --- |
|  | **Empa + SoC** | **SoC** | **Difference** | **Empa + SoC** | **SoC** | **Difference** | **Empa + SoC** | **SoC** | **Difference** |
| **Life-Years (LYs)** | | | | | | | | | |
| ***LYs, Total*** | ***5.81*** | ***5.62*** | ***0.18*** | ***5.96*** | ***5.77*** | ***0.19*** | ***6.11*** | ***5.91*** | ***0.20*** |
| KCCQ-CSS 1^st^ Quartile (0 to <55) | 0.87 | 0.94 | -0.06 | 0.89 | 0.96 | -0.06 | 0.92 | 0.98 | -0.06 |
| KCCQ-CSS 2^nd^ Quartile (55 to <75) | 1.16 | 1.12 | 0.03 | 1.19 | 1.15 | 0.03 | 1.21 | 1.18 | 0.04 |
| KCCQ-CSS 3^rd^ Quartile (75 to <90) | 1.56 | 1.57 | -0.01 | 1.60 | 1.61 | -0.01 | 1.64 | 1.65 | -0.01 |
| KCCQ-CSS 4^th^ Quartile (90 to 100) | 2.22 | 2.00 | 0.22 | 2.28 | 2.05 | 0.23 | 2.34 | 2.11 | 0.23 |
| **Quality-Adjusted Life-Years (QALYs)** | | | | | | | | | |
| ***QALYs, Total*** | ***3.76*** | ***3.57*** | ***0.19*** | ***4.51*** | ***4.28*** | ***0.23*** | ***3.98*** | ***3.77*** | ***0.21*** |
| KCCQ-CSS 1^st^ Quartile (0 to <55), Total | 0.37 | 0.39 | -0.01 | 0.47 | 0.48 | -0.02 | 0.34 | 0.36 | -0.01 |
| From State Occupancy | 0.45 | 0.49 | -0.03 | 0.56 | 0.60 | -0.04 | 0.43 | 0.46 | -0.03 |
| From Transient Events | -0.08 | -0.10 | 0.02 | -0.10 | -0.12 | 0.02 | -0.08 | -0.10 | 0.02 |
| KCCQ-CSS 2^nd^ Quartile (55 to <75), Total | 0.67 | 0.64 | 0.03 | 0.81 | 0.78 | 0.04 | 0.67 | 0.64 | 0.03 |
| From State Occupancy | 0.74 | 0.72 | 0.02 | 0.89 | 0.87 | 0.03 | 0.74 | 0.72 | 0.02 |
| From Transient Events | -0.07 | -0.08 | 0.01 | -0.08 | -0.09 | 0.01 | -0.07 | -0.08 | 0.01 |
| KCCQ-CSS 3^rd^ Quartile (75 to <90), Total | 1.05 | 1.05 | 0.00 | 1.26 | 1.26 | 0.00 | 1.12 | 1.12 | 0.00 |
| From State Occupancy | 1.11 | 1.11 | -0.01 | 1.33 | 1.34 | -0.01 | 1.18 | 1.19 | -0.01 |
| From Transient Events | -0.06 | -0.06 | 0.01 | -0.07 | -0.08 | 0.01 | -0.06 | -0.07 | 0.01 |
| KCCQ-CSS 4^th^ Quartile (90 to 100), Total | 1.67 | 1.49 | 0.17 | 1.97 | 1.76 | 0.21 | 1.84 | 1.65 | 0.19 |
| From State Occupancy | 1.72 | 1.55 | 0.17 | 2.03 | 1.83 | 0.20 | 1.90 | 1.71 | 0.19 |
| From Transient Events | -0.05 | -0.06 | 0.00 | -0.06 | -0.07 | 0.00 | -0.05 | -0.06 | 0.00 |
| **Clinical Outcomes** | | | | | | | | | |
| ***HF Rates, Total*** | ***1.2306*** | ***1.4042*** | ***-0.1735*** | ***1.2336*** | ***1.4069*** | ***-0.1733*** | ***1.2339*** | ***1.4072*** | ***-0.1733*** |
| KCCQ-CSS 1^st^ Quartile (0 to <55) | 0.3900 | 0.4689 | -0.0789 | 0.3908 | 0.4696 | -0.0788 | 0.3909 | 0.4697 | -0.0788 |
| KCCQ-CSS 2^nd^ Quartile (55 to <75) | 0.3217 | 0.3597 | -0.0380 | 0.3224 | 0.3604 | -0.0380 | 0.3224 | 0.3604 | -0.0380 |
| KCCQ-CSS 3^rd^ Quartile (75 to <90) | 0.2686 | 0.3108 | -0.0422 | 0.2694 | 0.3115 | -0.0421 | 0.2695 | 0.3116 | -0.0421 |
| KCCQ-CSS 4^th^ Quartile (90 to 100) | 0.2503 | 0.2647 | -0.0145 | 0.2510 | 0.2654 | -0.0144 | 0.2511 | 0.2655 | -0.0144 |
| ***CV Death (%)*** | ***69.1%*** | ***69.8%*** | ***-0.7%*** | ***69.3%*** | ***70.0%*** | ***-0.7%*** | ***69.3%*** | ***70.0%*** | ***-0.7%*** |
| KCCQ-CSS 1^st^ Quartile (0 to <55) | 23.8% | 25.6% | -1.8% | 23.9% | 25.7% | -1.8% | 23.9% | 25.7% | -1.8% |
| KCCQ-CSS 2^nd^ Quartile (55 to <75) | 16.1% | 15.8% | 0.2% | 16.1% | 15.9% | 0.2% | 16.1% | 15.9% | 0.2% |
| KCCQ-CSS 3^rd^ Quartile (75 to <90) | 13.3% | 13.6% | -0.3% | 13.3% | 13.6% | -0.3% | 13.3% | 13.6% | -0.3% |
| KCCQ-CSS 4^th^ Quartile (90 to 100) | 15.9% | 14.8% | 1.1% | 16.0% | 14.9% | 1.1% | 16.0% | 14.9% | 1.1% |
| ***Non-CV Death*** | ***30.8%*** | ***30.1%*** | ***0.7%*** | ***30.6%*** | ***29.9%*** | ***0.7%*** | ***30.6%*** | ***29.9%*** | ***0.7%*** |
| KCCQ-CSS 1^st^ Quartile (0 to <55) | 9.1% | 9.5% | -0.4% | 9.1% | 9.5% | -0.4% | 9.1% | 9.5% | -0.4% |
| KCCQ-CSS 2^nd^ Quartile (55 to <75) | 7.7% | 7.4% | 0.3% | 7.7% | 7.4% | 0.3% | 7.7% | 7.4% | 0.3% |
| KCCQ-CSS 3^rd^ Quartile (75 to <90) | 5.9% | 5.8% | 0.0% | 5.8% | 5.7% | 0.0% | 5.8% | 5.7% | 0.0% |
| KCCQ-CSS 4^th^ Quartile (90 to 100) | 8.1% | 7.4% | 0.7% | 8.0% | 7.3% | 0.7% | 8.0% | 7.3% | 0.7% |
| **Costs** | | | | | | | | | |
| ***Costs, Total*** | ***£16,661*** | ***£15,475*** | ***£1,185*** | ***€24,319*** | ***€22,549*** | ***€1,770*** | ***€21,726*** | ***€20,542*** | ***€1,183*** |
| KCCQ-CSS 1^st^ Quartile (0 to <55) | £3,448 | £3,668 | -£220 | €4,980 | €5,272 | -€291 | €4,550 | €4,907 | -€357 |
| KCCQ-CSS 2^nd^ Quartile (55 to <75) | £3,592 | £3,364 | £228 | €5,209 | €4,864 | €345 | €4,728 | €4,509 | €219 |
| KCCQ-CSS 3^rd^ Quartile (75 to <90) | £4,127 | £3,900 | £226 | €6,037 | €5,703 | €334 | €5,382 | €5,176 | €207 |
| KCCQ-CSS 4^th^ Quartile (90 to 100) | £5,494 | £4,543 | £951 | €8,093 | €6,711 | €1,382 | €7,066 | €5,951 | €1,115 |

Abbreviations: CV = cardiovascular; Empa = empagliflozin; HF = heart failure; KCCQ-CSS = Kansas City Cardiomyopathy Questionnaire Clinical Summary Score; LY = life-year; QALY = quality-adjusted life-year; SoC = standard of care; UK = United Kingdom

## Section S9. Deterministic Sensitivity Analysis

**Table S24.** **Deterministic sensitivity analysis scenarios and results for the United Kingdom**

| **Parameter** | **Base-case value** | **Alternate value** | **£/QALY** | |
| --- | --- | --- | --- | --- |
|  |  |  | **Low** | **High** |
| General model settings |  |  |  |  |
| Time horizon | Lifetime | 10 years | £6,461 | - |
|  |  | 20 years | £6,178 | - |
| Discount rate, cost | 3.5% | 0% to 5% | £7,434 | £5,729 |
| Discount rate, health | 3.5% | 0% to 5% | £4,945 | £6,680 |
| Clinical parameters |  |  |  |  |
| CV and all-cause death distributions | Weibull | Exponential | £6,054 | - |
|  |  | Lognormal | £6170 | - |
|  |  | Log-logistic | £6,145 | - |
|  |  | Generalised gamma | £6,154 | - |
|  |  | Gompertz | £6,751 | - |
| CV and all-cause death | Adjust with UK life tables | No adjustment | £6,145 | - |
| Treatment effect on CV death | Risk equation coefficient: -0.059 | Risk equation coefficient: 0 | £6,489 | - |
| Treatment effect on all-cause death | Risk equation coefficient: -0.044 | Risk equation coefficient: 0 | £6,725 | - |
| Treatment effect on HHF | Risk equation coefficient: -0.325 | Risk equation coefficient: 0 | £11,258 | - |
| Treatment discontinuation distribution | Exponential | Weibull | £6,197 | - |
| Treatment discontinuation inclusion | Include | Exclude | £6,468 | - |
| Utility age-adjustment | Yes | No | £5,700 | - |
| Utility by health state |  |  |  |  |
| KCCQ-CSS: 0 to <55 (Q1) | 0.520 | 0.512 to 0.528 | £6,168 | £6,168 |
| KCCQ-CSS: 55 to <75 (Q2) | 0.637 | 0.631 to 0.643 | £6,158 | £6,146 |
| KCCQ-CSS: 75 to <90 (Q3) | 0.710 | 0.704 to 0.716 | £6,150 | £6,154 |
| KCCQ-CSS: 90 to 100 (Q4) | 0.774 | 0.768 to 0.780 | £6,194 | £6,111 |
| Utility decrements |  |  |  |  |
| HHF decrement | -0.246 | -0.136 to -0.376 | £6,787 | £5,538 |
| AE decrement^a^ |  |  |  |  |
| Urinary tract infection | -0.025 | 0.000 to -0.99 | £6,143 | £6,163 |
| Genital mycotic infection | -0.058 | -0.008 to -0.155 |  |  |
| Acute renal failure | -0.010 | 0.000 to -0.045 |  |  |
| Hepatic injury | -0.016 | 0.000 to -0.069 |  |  |
| Volume depletion | -0.018 | -0.001 to -0.055 |  |  |
| Hypotension | -0.025 | -0.016 to -0.036 |  |  |
| Hypoglycaemic event | -0.048 | -0.006 to -0.131 |  |  |
| Bone fracture | -0.164 | -0.101 to -0.240 |  |  |
| Costs |  |  |  |  |
| Drug acquisition cost source | MIMS | eMIT | £6,099 | - |
| Empagliflozin cost per pack | £37 | £29 to £44 | £4,615 | £7,689 |
| HHF cost | £3,072 | £2,457 to £3,686 | £6,674 | £5,630 |
| CV death cost | £4,146 | £3,317 to £4,976 | £6,196 | £6,107 |
| CV death cost excluded | £4,146 | £0 | £6,375 | £6,152 |
| AE management cost^a^ |  |  |  |  |
| Urinary tract infection | £40 | £32 to £48 | £6,156 | £6,147 |
| Genital mycotic infection | £40 | £32 to £48 |  |  |
| Acute renal failure | £1,906 | £1,524 to £2,287 |  |  |
| Hepatic injury | £1,274 | £1,019 to £1,529 |  |  |
| Volume depletion | £40 | £32 to £48 |  |  |
| Hypotension | £40 | £32 to £48 |  |  |
| Hypoglycaemic event | £627 | £501 to £752 |  |  |
| Bone fracture | £2,710 | £2,168 to £3,252 |  |  |
| Disease management cost |  |  |  |  |
| Unit cost of disease management^a^ |  |  |  |  |
| Unit cost: GP Visit | £40 | £32 to £48 | £5,977 | £6,327 |
| Unit cost: Cardiologist visit | £140 | £112 to £168 |  |  |
| Unit cost: A&E referral | £154 | £123 to £184 |  |  |
| KCCQ-CSS: 0 to <55 (Q1) | £77 | £62 to £93 | £6,213 | £6,091 |
| KCCQ-CSS: 55 to <75 (Q2) | £77 | £62 to £93 | £6,120 | £7,689 |
| KCCQ-CSS: 75 to <90 (Q3) | £77 | £62 to £93 | £6,161 | £6,142 |
| KCCQ-CSS: 90 to 100 (Q4) | £77 | £62 to £93 | £5,938 | £6,168 |

^a^ Multi-way scenario

Abbreviations: A&E = accident and emergency; AE = adverse event; CV = cardiovascular; eMIT = electronic market information tool; GP = general practitioner; HHF = heart failure for worsening heart failure; KCCQ-CSS = Kansas City Cardiomyopathy Questionnaire Clinical Symptom Score; MIMS = Monthly Index of Medical Specialties; Q = quartile; QALY = quality-adjusted life year; UK = United Kingdom

**Table S25. Deterministic sensitivity analysis scenarios and results for Spain**

| **Parameter** | **Base-case value** | **Alternate value** | **€/QALY** | |
| --- | --- | --- | --- | --- |
|  |  |  | **Low** | **High** |
| General model settings |  |  |  |  |
| Time horizon | Lifetime | 10 years | €8,192 | - |
|  |  | 20 years | €7,780 | - |
| Discount rate, cost | 3.0% | 0% to 5% | €9,112 | €7,031 |
| Discount rate, health | 3.0% | 0% to 5% | €6,383 | €8,658 |
| Clinical parameters |  |  |  |  |
| CV and all-cause death distributions | Weibull | Exponential | €7,564 | - |
|  |  | Lognormal | €7,698 | - |
|  |  | Log-logistic | €7,687 | - |
|  |  | Generalised gamma | €7,739 | - |
|  |  | Gompertz | €8,589 | - |
| CV and all-cause death | Adjust with Spanish life tables | No adjustment | €7,731 | - |
| Treatment effect on CV death | Risk equation coefficient: -0.059 | Risk equation coefficient: 0 | €8,170 | - |
| Treatment effect on all-cause death | Risk equation coefficient: -0.044 | Risk equation coefficient: 0 | €8,561 | - |
| Treatment effect on HHF | Risk equation coefficient: -0.325 | Risk equation coefficient: 0 | €13,520 | - |
| Treatment discontinuation distribution | Exponential | Weibull | €7,789 | - |
| Treatment discontinuation inclusion | Include | Exclude | €8,104 | - |
| Utility age-adjustment | Yes | No | €7,843 | - |
| Utility by health state |  |  |  |  |
| KCCQ-CSS: 0 to <55 (Q1) | 0.629 | 0.620 to 0.637 | €7,867 | €7,754 |
| KCCQ-CSS: 55 to <75 (Q2) | 0.754 | 0.747 to 0.761 | €7,744 | €7,728 |
| KCCQ-CSS: 75 to <90 (Q3) | 0.832 | 0.826 to 0.838 | €7,734 | €7,738 |
| KCCQ-CSS: 90 to 100 (Q4) | 0.891 | 0.885 to 0.897 | €7,784 | €7,689 |
| Utility decrements |  |  |  |  |
| HHF decrement | -0.291 | -0.228 to -0.353 | €8,428 | €7,077 |
| AE decrement^a^ |  |  |  |  |
| Urinary tract infection | -0.025 | 0.000 to -0.099 | €7,727 | €7,749 |
| Genital mycotic infection | -0.053 | -0.005 to -0.152 |  |  |
| Acute renal failure | -0.014 | -0.001 to -0.047 |  |  |
| Hepatic injury | -0.011 | 0.000 to -0.068 |  |  |
| Volume depletion | -0.015 | -0.001 to -0.054 |  |  |
| Hypotension | -0.025 | -0.016 to -0.036 |  |  |
| Hypoglycaemic event | -0.041 | -0.003 to -0.128 |  |  |
| Bone fracture | -0.170 | -0.106 to -0.245 |  |  |
| Costs |  |  |  |  |
| Drug acquisition cost source | Retail price including VAT | Ex-factory price | €4,073 |  |
| Empagliflozin cost per pack | €55 | €44 to €67 | €5,878 | €9,594 |
| HHF cost | €3,814 | €3,051 to €4,577 | €8,286 | €7,186 |
| CV death cost | €6,276 | € 5,020 to €7,531 | €7,791 | €7,681 |
| CV death cost excluded | €6,276 | £0 | €8,009 | €7,736 |
| AE management cost^a^ |  |  |  |  |
| Urinary tract infection | €57 | €45 to €68 | €7,750 | €7,722 |
| Genital mycotic infection | €57 | €45 to €68 |  |  |
| Acute renal failure | €4,243 | €3,395 to €5,092 |  |  |
| Hepatic injury | €2,709 | €2,167 to €3,251 |  |  |
| Volume depletion | €57 | €45 to €68 |  |  |
| Hypotension | €57 | €45 to €68 |  |  |
| Hypoglycaemic event | €1,461 | €1,169 to €1,753 |  |  |
| Bone fracture | €5,042 | €4,033 to €6,050 |  |  |
| Disease management cost |  |  |  |  |
| Unit cost of disease management^a^ |  |  |  |  |
| Unit cost: GP Visit | €57 | €45 to €68 | €7,627 | €7,845 |
| Unit cost: Cardiologist visit | €115 | €92 to €139 |  |  |
| Unit cost: A&E referral | €196 | €157 to €235 |  |  |
| KCCQ-CSS: 0 to <55 (Q1) | €55 | €44 to €66 | €7,773 | €7,699 |
| KCCQ-CSS: 55 to <75 (Q2) | €55 | €44 to €66 | €7,716 | €7,756 |
| KCCQ-CSS: 75 to <90 (Q3) | €55 | €44 to €66 | €7,741 | €7,731 |
| KCCQ-CSS: 90 to 100 (Q4) | €55 | €44 to €66 | €7,605 | €7,867 |

^a^ Multi-way scenario

Abbreviations: A&E = accident and emergency; AE = adverse event; CV = cardiovascular; GP = general practitioner; HHF = heart failure for worsening heart failure; KCCQ-CSS = Kansas City Cardiomyopathy Questionnaire Clinical Symptom Score; Q = quartile; QALY = quality-adjusted life year; VAT = value-added tax

**Table S26. Deterministic sensitivity analysis scenarios and results for France**

| **Parameter** | **Base-case value** | **Alternate value** | **€/QALY** | |
| --- | --- | --- | --- | --- |
|  |  |  | **Low** | **High** |
| General model settings |  |  |  |  |
| Time horizon | Lifetime | 10 years | €5,505 | - |
|  |  | 20 years | €5,511 | - |
| Discount rate, cost | 2.5% | 0% to 5% | €6,462 | €4,790 |
| Discount rate, health | 2.5% | 0% to 5% | €4,705 | €6,330 |
| Clinical parameters |  |  |  |  |
| CV and all-cause death distributions | Weibull | Exponential | €5,455 | - |
|  |  | Lognormal | €5,404 | - |
|  |  | Log-logistic | €5,434 | - |
|  |  | Generalised gamma | €5,508 | - |
|  |  | Gompertz | €5,665 | - |
| CV and all-cause death | Adjust with French life tables | No adjustment | €5,510 | - |
| Treatment effect on CV death | Risk equation coefficient: -0.059 | Risk equation coefficient: 0 | €5,790 | - |
| Treatment effect on all-cause death | Risk equation coefficient: -0.044 | Risk equation coefficient: 0 | €5,467 | - |
| Treatment effect on HHF | Risk equation coefficient: -0.325 | Risk equation coefficient: 0 | €11,810 | - |
| Treatment discontinuation distribution | Exponential | Weibull | €5,557 | - |
| Treatment discontinuation inclusion | Include | Exclude | €5,829 | - |
| Utility age-adjustment | Yes | No | €5,347 | - |
| Utility by health state |  |  |  |  |
| KCCQ-CSS: 0 to <55 (Q1) | 0.464 | 0.455 to 0.474 | €5,495 | €5,527 |
| KCCQ-CSS: 55 to <75 (Q2) | 0.610 | 0.602 to 0.618 | €5,518 | €5,503 |
| KCCQ-CSS: 75 to <90 (Q3) | 0.721 | 0.714 to 0.728 | €5,509 | €5,512 |
| KCCQ-CSS: 90 to 100 (Q4) | 0.810 | 0.803 to 0.817 | €5,554 | €5,468 |
| Utility decrements |  |  |  |  |
| HHF decrement | -0.240 | -0.168 to -0.312 | €6,104 | €4,922 |
| AE decrement^a^ |  |  |  |  |
| Urinary tract infection | -0.025 | 0.000 to -0.099 | €5,504 | €5,524 |
| Genital mycotic infection | -0.052 | -0.002 to -0.170 |  |  |
| Acute renal failure | -0.011 | 0.000 to -0.051 |  |  |
| Hepatic injury | -0.018 | 0.000 to -0.071 |  |  |
| Volume depletion | -0.017 | -0.001 to -0.054 |  |  |
| Hypotension | -0.025 | -0.016 to -0.036 |  |  |
| Hypoglycaemic event | -0.055 | -0.006 to -0.152 |  |  |
| Bone fracture | -0.148 | -0.086 to -0.225 |  |  |
| Costs |  |  |  |  |
| Drug acquisition cost source | NA | NA |  |  |
| Empagliflozin cost per pack | €38 | €31 to €46 | €4,123 | €6,898 |
| HHF cost | €4,968 | €3,974 to €5,961 | €6,281 | €4,741 |
| CV death cost | €3,764 | €3,011 to €4,516 | €5,544 | €5,477 |
| CV death cost excluded | €3,764 | £0 | €5,679 | €5,511 |
| AE management cost^a^ |  |  |  |  |
| Urinary tract infection | €36 | €29 to €43 | €5,524 | €5,498 |
| Genital mycotic infection | €36 | €29 to €43 |  |  |
| Acute renal failure | €3,739 | €2,991 to €4,486 |  |  |
| Hepatic injury | €1,055 | €844 to €1,266 |  |  |
| Volume depletion | €36 | €29 to €43 |  |  |
| Hypotension | €36 | €29 to €43 |  |  |
| Hypoglycaemic event | €2,319 | €1,855 to €2,783 |  |  |
| Bone fracture | €3,352 | €2,682 to €4,022 |  |  |
| Disease management cost |  |  |  |  |
| Unit cost of disease management^a^ |  |  |  |  |
| Unit cost: GP Visit | €36 | €29 to €43 | €5,406 | €5,615 |
| Unit cost: Cardiologist visit | €243 | €194 to €291 |  |  |
| Unit cost: A&E referral | €151 | €121 to €181 |  |  |
| KCCQ-CSS: 0 to <55 (Q1) | €48 | €38 to €57 | €5,545 | €5,477 |
| KCCQ-CSS: 55 to <75 (Q2) | €48 | €38 to €57 | €5,491 | €5,530 |
| KCCQ-CSS: 75 to <90 (Q3) | €48 | €38 to €57 | €5,515 | €5,506 |
| KCCQ-CSS: 90 to 100 (Q4) | €48 | €38 to €57 | €5,387 | €5,634 |

^a^ Multi-way scenario

Abbreviations: A&E = accident and emergency; AE = adverse event; CV = cardiovascular; GP = general practitioner; HHF = heart failure for worsening heart failure; KCCQ-CSS = Kansas City Cardiomyopathy Questionnaire Clinical Symptom Score; Q = quartile; QALY = quality-adjusted life year

## Section S10. Probabilistic Sensitivity Analysis

**Table S27. Parameters and distributions for probabilistic sensitivity analysis**

| **Parameter** | **Probabilistic distribution** |
| --- | --- |
| Statistical models for HHF, CV death, all-cause death, and treatment discontinuation^a^ | Correlated draws from multivariate normal distributions from Cholesky decomposition of covariance matrices |
| KCCQ-CSS transition probabilities^b^ | Dirichlet |
| AE rates^c^ | Gamma |
| Utility by health state^d^ | Beta |
| Utility decrements^d^ | Beta |
| Clinical event management costs^e^ | Gamma |
| Disease management costs^e^ | Gamma |

^a^ Statistical models are shown in Section S3.

^b^ Transition probabilities are shown in Section S3.

^c^ AE rates are shown in Section S4.

^d^ Utility values are shown in Section S5.

|^e^ Costs are shown in Section S6.

Abbreviations: AE = adverse event; CV = cardiovascular; HHF = heart failure for worsening heart failure; KCCQ-CSS = Kansas City Cardiomyopathy Questionnaire Clinical Symptom Score

**Fig. S1 Cost-effectiveness acceptability curves**

| ***UK***  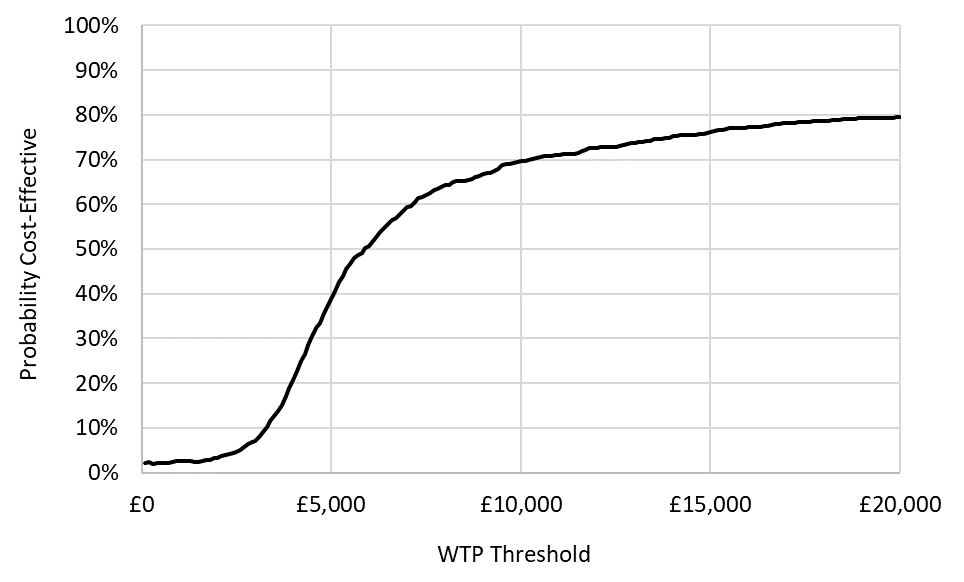 | ***Spain***  **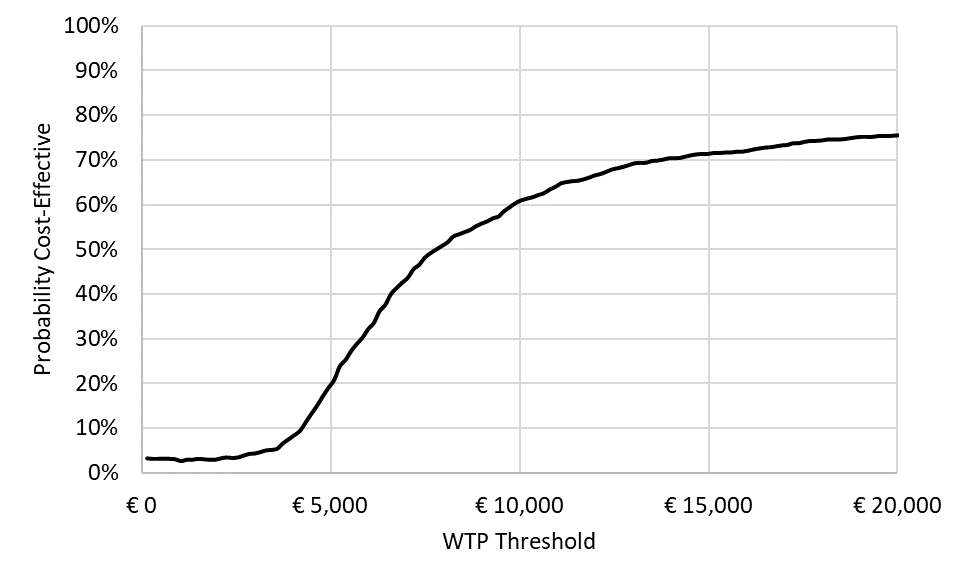** |
| --- | --- |
| ***France***  ***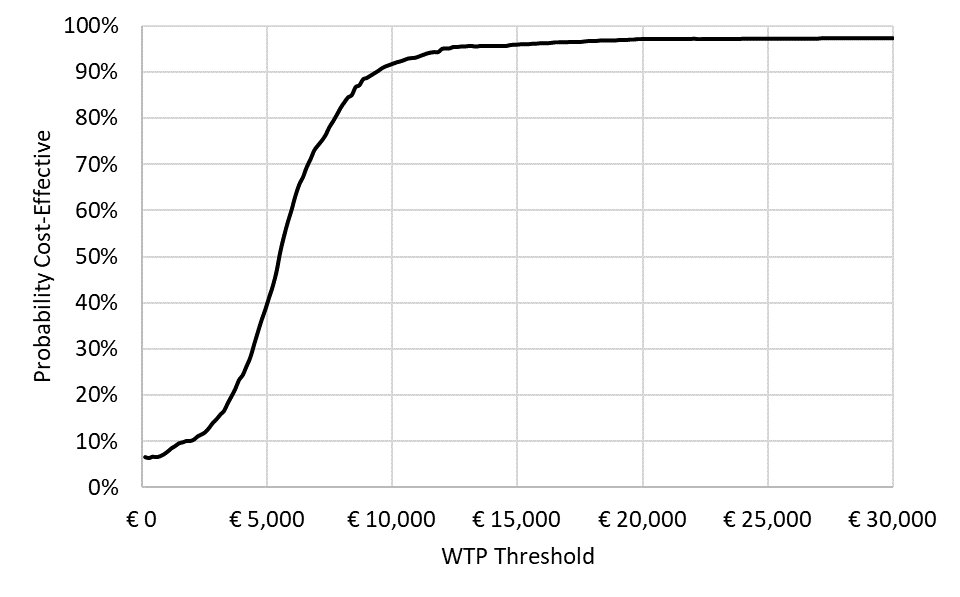*** |  |

Abbreviations: UK = United Kingdom; WTP = willingness-to-pay

## References

1. van Hout B, Janssen MF, Feng YS, et al. Interim scoring for the EQ-5D-5L: mapping the EQ-5D-5L to EQ-5D-3L value sets. *Value Health*. Jul-Aug 2012;15(5):708-15. doi:10.1016/j.jval.2012.02.008
2. National Institute for Health and Care Excellence (NICE), Latimer N. NICE DSU technical support document 14: survival analysis for economic evaluations alongside clinical trials-extrapolation with patient-level data. Accessed July 2020, nicedsu.org.uk/wp-content/uploads/2016/03/NICE-DSU-TSD-Survival-analysis.updated-March-2013.v2.pdf
3. Sullivan PW, Slejko JF, Sculpher MJ, Ghushchyan V. Catalogue of EQ-5D scores for the United Kingdom. *Med Decis Making*. Nov-Dec 2011;31(6):800-4. doi:10.1177/0272989X11401031
4. Szende A, Janssen B, Cabases J, eds. *Self-Reported Population Health: An International Perspective based on EQ-5D*. Springer; 2014.
5. Janssen B, Szende A. Population Norms for the EQ-5D. In: Szende A, Janssen B, Cabases J, eds. *Self-Reported Population Health: An International Perspective based on EQ-5D*. Springer; 2014:19-30.
6. Sullivan PW, Ghushchyan VH. EQ-5D Scores for Diabetes-Related Comorbidities. *Value Health*. Dec 2016;19(8):1002-1008. doi:10.1016/j.jval.2016.05.018
7. Sullivan PW, Ghushchyan V. Preference-Based EQ-5D index scores for chronic conditions in the United States. *Med Decis Making*. Jul-Aug 2006;26(4):410-20. doi:10.1177/0272989X06290495
8. Monthly Index of Medical Specialties. Drug Database. Accessed September 1, 2021. https://www.mims.co.uk/
9. National Health Service. National Schedule of NHS Costs - Year 2018-19. Accessed November 1, 2020. https://www.england.nhs.uk/national-cost-collection/
10. Alva ML, Gray A, Mihaylova B, Leal J, Holman RR. The impact of diabetes-related complications on healthcare costs: new results from the UKPDS (UKPDS 84). *Diabet Med*. Apr 2015;32(4):459-66. doi:10.1111/dme.12647
11. Curtis LA, Burns A. Unit Costs of Health and Social Care 2019 Personal Social Services Research Unit (PSSRU). Accessed November 1, 2020. https://www.pssru.ac.uk/project-pages/unit-costs/unit-costs-2019/
12. McMurray JJV, Trueman D, Hancock E, et al. Cost-effectiveness of sacubitril/valsartan in the treatment of heart failure with reduced ejection fraction. *Heart*. Jun 2018;104(12):1006-1013. doi:10.1136/heartjnl-2016-310661
13. Consejo General de Colegios Farmaceuticos. BOT PLUS web. Accessed July 1, 2021. https://botplusweb.portalfarma.com/botplus.aspx
14. Ministerio de Sanidad Consumo y Bienestar Social. Registro de altas - Categoría CIE-10 – CMBD. Accessed February, 2021. https://pestadistico.inteligenciadegestion.mscbs.es/
15. Decreto Legislativo 1/2005, de 25 de Febrero por el que se Aprueba el Texto Refundido de la Ley de Tasas de la Generalitat. Accessed September 1, 2021. http://www.san.gva.es/documents/151744/2847194/LEY_DE_TASAS_2015.docx
16. Boletín Oficial de Castilla y León. Decreto 25/2010, de 17 de junio, sobre precios públicos por actos asistenciales y servicios sanitarios prestados por la Gerencia Regional de Salud. Accessed September 2021, https://www.saludcastillayleon.es/transparencia/es/transparencia/informacion-datos-publicos/gestion-economica/coste-servicios/precios-publicos-actos-asistenciales-servicios-sanitarios.ficheros/1199560-01%20ANEXO%20PRECIOS%20P%C3%9ABLICOS.pdf
17. Boletín Oficial de la Comunidad de Madrid. Precios publicos por la prestacion de los servicios y actividades de naturaleza sanitaria. Accessed September 1, 2021. http://www.madrid.org/cs/Satellite?blobcol=urldata&blobheader=application%2Fpdf&blobheadername1=Content-Disposition&blobheadervalue1=filename%3DPrecios+p%C3%BAblicos-79942.pdf&blobkey=id&blobtable=MungoBlobs&blobwhere=1352936719948&ssbinary=true
18. Diari Oficial de la Generalitat de Catalunya. ORDEN SLT/71/2020, de 2 de junio, por la que se regulan los supuestos y conceptos facturables y se aprueban los precios públicos correspondientes a los servicios que presta el Instituto Catalán de la Salud. Accessed September 1, 2021. https://portaldogc.gencat.cat/utilsEADOP/PDF/8153/1799007.pdf
19. Osakidetza. Tarifas para Facturacion de Servicios Sanitarios y Docentes de Osakidetza para el Ano 2020. Accessed September 1, 2021. https://www.osakidetza.euskadi.eus/contenidos/informacion/osk_servic_para_empresas/es_def/adjuntos/LIBRO-DE-TARIFAS_2020_osakidetza.pdf
20. Servicio Andaluz de Salud. Precios publicos. Accessed September 1, 2021. https://www.sspa.juntadeandalucia.es/servicioandaluzdesalud/profesionales/recursos-para-profesionales/precios-publicos
21. Escobar C, Varela L, Palacios B, et al. Costs and healthcare utilisation of patients with heart failure in Spain. *BMC Health Serv Res*. Oct 20 2020;20(1):964. doi:10.1186/s12913-020-05828-9
22. Legifrance. Avis relatif à l'avenant n° 20 à la convention nationale du 4 avril 2012 organisant les rapports entre les pharmaciens titulaires d'officine et l'assurance maladie. Accessed September 1, 2021. https://www.legifrance.gouv.fr/jo_pdf.do?id=JORFTEXT000041931400
23. European Society of Cardiology. ESC Guidelines for the diagnosis and treatment of acute and chronic heart failure. Accessed September 1, 2021. https://www.escardio.org/Guidelines/Clinical-Practice-Guidelines/Acute-and-Chronic-Heart-Failure
24. Medicaments BdDPd. IVABRADINE ARROW 7,5 mg, comprimé pelliculé - Résumé des caractéristiques du produit. Accessed September 1, 2021. https://base-donnees-publique.medicaments.gouv.fr/affichageDoc.php?specid=61432512&typedoc=R
25. Haute Autorité de Santé. Entresto efficiency report. Accessed August 19, 2021. https://www.has-sante.fr/upload/docs/application/pdf/2017-12/entresto_12042016_avis_efficience.pdf
26. Agence technique de l'information sur l'hospitalisation. ENC/T2A: French case-mix-based prospective payment system. Accessed November 23, 2021. https://www.atih.sante.fr/tarifs-mco-et-had
27. Chouaid C, Loirat D, Clay E, et al. Cost analysis of adverse events associated with non-small cell lung cancer management in France. *Clinicoecon Outcomes Res*. 2017;9:443-449. doi:10.2147/CEOR.S138963
28. Torreton E, Vandebrouck T, Emiel P, Detournay B. Cost of Inpatient Management of Hypoglycaemia in France. *Value Health*. 2013;16(7):A436.
29. Sécurité Sociale l'Assurance Maladie. French healthcare insurance: Activité globale et prescriptions des professionnels de santé libéraux. Accessed November 23, 2021. https://assurance-maladie.ameli.fr/etudes-et-donnees/entree-par-theme/professionnels-de-sante-liberaux/activite-globale-et-prescriptions/activite-globale-prescriptions-professionnels-sante-liberaux
30. Sécurité Sociale l'Assurance Maladie. French healthcare insurance: Honoraires des professionnels de santé libéraux. Accessed November 23, 2021. https://assurance-maladie.ameli.fr/etudes-et-donnees/entree-par-theme/professionnels-de-sante-liberaux/honoraires/honoraires-professionnels-sante-liberaux
31. Sicras-Mainar A, Sicras-Navarro A, Palacios B, Varela L, Delgado JF. Epidemiology and treatment of heart failure in Spain: the HF-PATHWAYS study. *Rev Esp Cardiol (Engl Ed)*. Jan 2022;75(1):31-38. doi:10.1016/j.rec.2020.09.033
32. The Heart Failure Policy Network. Heart failure policy and practice in Europe. Accessed October 17, 2022. https://www.hfpolicynetwork.org/wp-content/uploads/Heart-failure-policy-and-practice-in-Europe-France.pdf
33. Escobar C, Varela L, Palacios B, et al. Clinical characteristics, management, and one-year risk of complications among patients with heart failure with and without type 2 diabetes in Spain. *Rev Clin Esp (Barc)*. Apr 2022;222(4):195-204. doi:10.1016/j.rceng.2021.04.005
